# Influence of Erythropoietin on Cognitive Performance during Experimental Hypoglycemia in Patients with Type 1 Diabetes Mellitus: A Randomized Cross-Over Trial

**DOI:** 10.1371/journal.pone.0059672

**Published:** 2013-04-05

**Authors:** Peter Lommer Kristensen, Ulrik Pedersen-Bjergaard, Troels Wesenberg Kjær, Niels Vidiendal Olsen, Flemming Dela, Jens Juul Holst, Jens Faber, Lise Tarnow, Birger Thorsteinsson

**Affiliations:** 1 Endocrinology Section, Department of Cardiology, Nephrology and Endocrinology, Hillerød Hospital, Hillerød, Denmark; 2 Clinic of Neurophysiology, The Neuroscience Centre, Rigshospitalet, Copenhagen University Hospital, Copenhagen, Denmark; 3 Department of Neuroanaesthesia, The Neuroscience Centre, Rigshospitalet, Copenhagen University Hospital, Copenhagen, Denmark; 4 Center for Health Aging, Department of Medical Sciences, Faculty of Health Sciences, University of Copenhagen, Denmark; 5 Department of Medical Physiology, Faculty of Health Sciences, University of Copenhagen, Copenhagen, Denmark; 6 Steno Diabetes Center, Gentofte, Denmark; 7 Department of Orthopaedics and Internal Medicine, Faculty of Health Sciences, University of Copenhagen, Copenhagen, Denmark; 8 Department of Neuroscience and Pharmacology, Faculty of Health Sciences, University of Copenhagen, Copenhagen, Denmark; 9 Department of Internal Medicin O, Herlev University Hospital, Herlev, Denmark; Tehran University of Medical Sciences, Iran (Republic of Islamic)

## Abstract

**Introduction:**

The incidence of severe hypoglycemia in type 1 diabetes has not decreased over the past decades. New treatment modalities minimizing the risk of hypoglycemic episodes and attenuating hypoglycemic cognitive dysfunction are needed. We studied if treatment with the neuroprotective hormone erythropoietin (EPO) enhances cognitive function during hypoglycemia.

**Materials and Methods:**

Eleven patients with type 1 diabetes, hypoglycemia unawareness and recurrent severe hypoglycemia completed the study. In a double-blind, randomized, balanced, cross-over study using clamped hypoglycemia they were treated with 40,000 IU of EPO or placebo administered intravenously six days before the two experiments. Cognitive function (primary endpoint), hypoglycemic symptoms, and counter-regulatory hormonal response were recorded.

**Results:**

Compared with placebo, EPO treatment was associated with a significant reduction in errors in the most complex reaction time task (−4.7 (−8.1 to −1.3), p = 0.01) and a less reaction time prolongation (−66 (−117 to −16) msec, p = 0.02). EPO treatment did not change performance in other measures of cognition. Hypoglycemic symptoms, EEG-changes, and counter-regulatory hormone concentrations did not differ between EPO and placebo treatment.

**Conclusion:**

In patients with type 1 diabetes and hypoglycemia unawareness, treatment with EPO is associated with a beneficial effect on cognitive function in a complex reaction time task assessing sustained attention/working memory. Hypoglycemic symptoms and hormonal responses were not changed by EPO treatment.

**Trial Registration:**

ClinicalTrials.gov NCT00615368

## Introduction

Risk of severe hypoglycemia represents a major problem in type 1 diabetes. The episodes are feared by patients [1] and relatives [2] due to loss of selfcare during the episodes and they remain a dominating obstacle in reaching glycemic targets [3]. Despite development of educational programs, insulin analogues and new insulin delivery systems, the frequency of severe hypoglycemia has not decreased over the past decades [4]–[7]. Therefore new treatment modalities should be considered. Neuroprotection during hypoglycemia may represent an alternative treatment strategy that could reduce the clinical consequences of unavoidable hypoglycemic episodes in daily life.

Erythropoietin (EPO) is a glycoprotein mainly produced in the kidney. Its role in erythropoiesis is well known. In recent years, a non-hematopoietic neuroprotective role has emerged for EPO in conditions with impaired substrate supply, e.g. during hypoxia [8]. EPO and its receptor are produced in the brain, and is increased in response to intracerebral metabolic stress such as acute brain hypoxia [9], [10]. EPO receptors are located in brain endothelial cells in rat and mouse [11]. At supraphysiological circulating concentrations EPO penetrates the intact human blood brain barrier [12]. *In vitro* studies suggest that EPO preserves cellular function during hypoglycemia [13]–[16]. In healthy adults high dose intravenous EPO treatment modulates neuronal processing and may improve cognitive function 7 days after administration of EPO [17], [18]. In accordance, a beneficial effect of intravenous EPO treatment on cognitive function in patients with chronic schizophrenia and cognitive decline exists [19]. We recently showed in patients with type 1 diabetes that the concentration of EPO increases modestly in response to hypoglycemia and that low baseline EPO levels may be associated with more pronounced cerebral dysfunction during experimental hypoglycemia than higher levels of EPO [20]. The aim of the present study is to test the effects of EPO treatment on cognitive function, hypoglycemic symptoms, counter-regulatory hormonal responses and cortical electrical activity during mild hypoglycemia in patients with type 1 diabetes.

## Materials and Methods

The protocol for this trial and supporting CONSORT checklist are available as supporting information; see Checklist S1, Protocol S1, and Protocol Danish S1.

### Subjects

**Figure 1:**
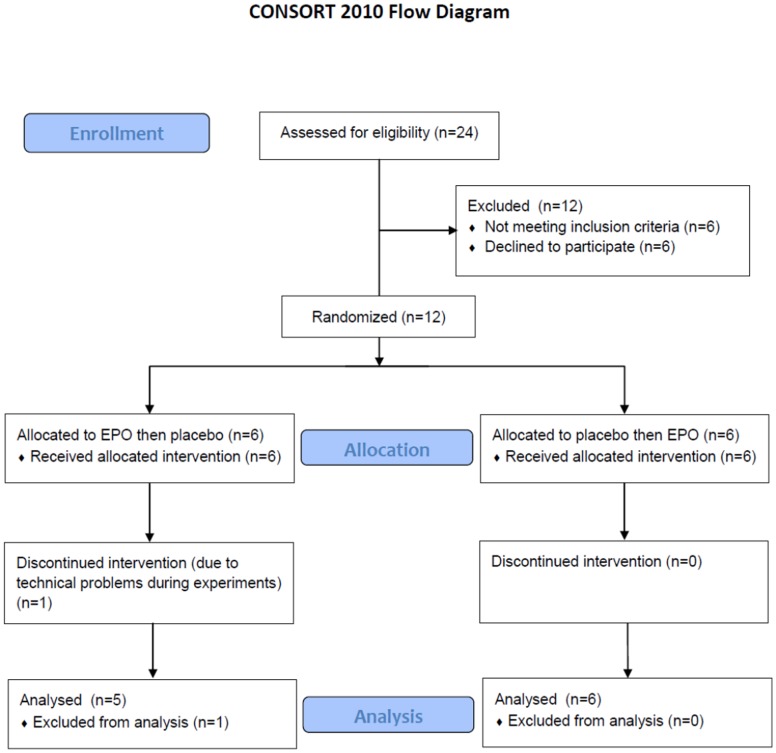
The Consort diagram.

Eleven subjects with type 1 diabetes were included (by Peter Lommer Kristensen (PLK)) from the outpatient clinics at Hillerød Hospital and Steno Diabetes Center. The participants were invited by letter from a group of patients identified as having high risk of severe hypoglycemia in an earlier study [21]. Criteria of inclusion were: Two or more episodes of severe hypoglycemia in the preceding year, hypoglycemia unawareness [22], age >18 years, duration of diabetes >5 years, weight >50 kg, negative pregnancy test and written informed consent. Criteria of exclusion were: Plasma creatinine concentration >100 µmol/l for males and >88 µmol/l for females, hemoglobin concentration >11 or <7 mmol/l, surgery within the last 6 weeks, history of cancer, thrombocytosis, stroke/transient ischemic attack, deep venous thrombosis/pulmonary embolism or myocardial infarction, epilepsy, heart failure (NYHA >1), or ischemic heart disease, treatment with ciclosporin, EPO (also previously) or beta receptor antagonists, pregnancy or problems with perception. The experiments were carried out from January 2009 to December 2009. Baseline characteristics are shown in Table 1 and Consort Diagram in Figure 1.

**Table 1:** Baseline characteristics.

Females/males, number	3/8
Age, years	53 (26–68)
Duration of diabetes, years	29 (5–44)
C-peptide^a^, pmol/l, median (range)	<0.2 (<0.2–0.9)
HbA1c, %	7.6 (6.9–9.0)
Daily insulin dose per kilo, IU/kg	0.61 (0.46–0.77)
Body mass index, kg/m^2^	24.8 (21.4–27.8)
Retinopathy, none/background/proliferative, number	3/6/2
Nephropathy, none/microalbuminuria/albuminuria, number	8/2/1
Plasma creatinine, µmol/l	68 (59–82)
Treated for hypertension, −/+, number	5/6
Total episodes (all subjects) of severe hypoglycemia in the week before the baseline visits, number (converted toepisodes/patient/year)	3 (7.1)
Total episodes (all subjects) of mild hypoglycemia in the week before the baseline visits, number (converted toepisodes/week/patient)	60 (2.7)

Baseline demographic and diabetes-related characteristics of 11 patients with type 1 diabetes. Numbers are means (range) unless otherwise stated.

a C-peptide at baseline visit. Detection limit 0.2 nmol/l.

### Design

The study is a double-blind, randomized, balanced, cross-over study evaluating the effect of 40,000 IU of intravenously administered erythropoietin (1 ml Eprex®, Janssen-Cilag, 40,000 IU/ml) or placebo (1 ml isotonic sodium chloride) on cognitive function (primary endpoint), hypoglycemic symptoms, counter-regulatory hormonal responses, and cortical electrical activity during experimental hypoglycemia (secondary endpoints). Six days before the hypoglycemic experiments, 1∶1 randomization (using randomization lists (done by PLK)) and injection of study medication or placebo were done by a study nurse not involved in endpoint registration. The interval of six days was chosen because Miskowiak et al showed that 40,000 IE of intravenously administered EPO lead to better performance in healthy subjects in a test of verbal fluency 7 days (but not at an earlier time point) after injection of EPO [17]. It was not possible for participants to visually distinguish between placebo and study medication. Randomization lists were not available for persons involved in endpoint registration and participants were not informed about the randomization sequence until the end of data analysis. A predefined wash-out period of at least 6 weeks was interposed between the two experiments. Median interval between experiments was 7 weeks (range 6–12). Sample size calculations (done on a calculator in Microsoft Excel) showed that approximately 10 subjects were needed (two-sided significance level of 5%, power of 80%) to detect a difference of 50 msec between the two days of intervention (EPO and placebo) in the reaction time tests (CalCAP). These calculations were based on earlier data from our research group showing that the standard error of the mean of reaction time during hypoglycemia was approximately 40 msec. The potential clinical relevance of a ∼50 msec deterioration of reaction time during hypoglycemia is not known. It may be very relevant if hypoglycemia occurs when driving a vehicle but far less relevant under other circumstances.

The Regional Committee on Biomedical Research Ethics and the Danish Medicines Agency approved the study. The study was conducted according to the principles of Good Clinical Practice (GCP), monitored by The GCP Unit at the University of Copenhagen, and registered on Clinicaltrials.gov (NCT00615368). The experiments were done at the Section of Endocrinology at Hillerød University Hospital, Denmark.

### Experimental protocol

#### 6 days before hypoglycemic clamp

Subjects attended the clinic, and information about demography, lifestyle and clinical characteristics was collected. A physical examination with focus on possible thromboembolic disease was performed. Blood pressure was measured twice after 10 min rest in a sitting position. Measures of peripheral and autonomic neuropathy were assessed and a 12-lead electrocardiogram (ECG) was visually assessed. State of hypoglycemia awareness was classified according to Pedersen-Bjergaard et al [22]. The frequencies of episodes of mild and severe hypoglycemia in the preceding week were recorded in a questionnaire. A sensor for continuous glucose monitoring (CGM) was mounted (Guardian® REAL-time, Medtronic Minimed, Northridge, USA). Hereafter, the subject was re-introduced to the experimental protocol and cognitive tests were trained twice to avoid a learning effect during the experiments. Finally, study medication was injected intravenously over four minutes. Subjects were instructed to live and eat as they used to do, to avoid any rigorous exercise, use of alcohol and psychoactive drugs, for the next week. Calibration of the CGM was done by the patient with the blood glucose meter CONTOUR® LINK (Bayer Health Care, Leverkusen, Germany).

#### 3 days before experiment

A new CGM sensor was inserted and a hypoglycemia alarm was activated. Because the CGM glucose value is delayed approximately 10 min compared to plasma glucose the alarm-point was set to 4.5 mmol/l. CGM glucose profiles for the last three days were downloaded to check if any insulin dosage changes should be done to decrease the risk of hypoglycemia until the experiment 3 days later. Finally, the subjects trained the cognitive function tests again.

##### Day of experiment

Subjects arrived at 8.00 a.m. in the laboratory after an overnight fast. Data from the CGM were downloaded. If hypoglycemia, defined by at least two consecutive CGM values below 3.5 mmol/l or one self-monitored blood glucose value below 3.5 mmol/l, had occurred during the preceding night, the experiment was postponed for at least 14 days. This was the case at one occasion. CGM warned the participants about decreasing blood glucose during the night before the experiment at three occasions before it actually occurred. In these cases the participants were instructed to eat a standardized meal of 10 grams of glucose and 35 g of dark bread. No participants had a CGM glucose >20 mmol/l during the night before the experiments.

The subject was introduced to the experimental setting and was equipped as follows: 1) One antecubital intravenous line for infusion of insulin and glucose and one line as distally as possible for blood sampling. Thereafter, the hand and arm were placed on and under heated pads to arterialize venous blood; 2) EEG (electroencephalography) cap, two ear lobe references and two precordial ECG leads connected to a digital EEG recorder (Cadwell, Kennewick, Washington, USA); 3) an automatic blood pressure unit (UA787, A&D Medical, Abingdon, UK). Subsequently, subjects trained the cognitive tests.

The experiment was carried out in a cyclic manner with a total of three identical cycles: A baseline cycle (euglycemia), a hypoglycemic cycle and a recovery cycle (euglycemia). In the start of each cycle arterialized venous blood was drawn. Subsequently, hypoglycemic symptoms were assessed followed by measurement of blood pressure and pulse rate. Hereafter, evaluation of cognitive function was done with the California Cognitive Assessment Package (CalCAP), a trail making test and the Stroop test. All cognitive testing was conducted in the same sequence by the same person (PLK). At the end of each cycle, 5 min of EEG recording was done with eyes closed. Finally, arterialized venous blood was drawn again.

### Hypoglycemic Clamp Procedure

Short-acting human insulin (Actrapid®, Novo Nordisk, Bagsværd, Denmark) mixed with heparinized plasma from the patient and isotonic saline was infused at a rate of 1.0 mU×kg body weight^−1^×min^−1^. Capillary blood glucose was measured bedside every 5 min and adjusted with a variable infusion of 20% glucose. The hypoglycemic target was 2.0–2.2 mmol/l. During the euglycemic cycles the target was 5.0 mmol/l.

### Hypoglycemia Symptom Scoring

Hypoglycemic symptoms were assessed by the The Edinburgh Hypoglycemia Scale, which is a questionnaire developed for experimentally induced hypoglycemia in which subjects must indicate the severity of 13 pre-defined hypoglycemic symptoms on a scale from one to seven [23].

### Cognitive Function Tests

#### California cognitive assessment package (CalCAP)

The Danish version of the Mini CalCAP test (E.N Miller, California Cognitive Assessment Package, Norland Software, Los Angeles, 1990) runs on a computer screen and consists of two different reaction time tasks with different complexity: 1) a choice reaction task (RT1) with reaction to a specific number (seven) and 2) a choice reaction task (RT2) with reaction to two identical numbers in a sequence (shifting numbers). The latter test is a 1-back task and includes the use of sustained attention/working memory.

#### Trail making test

The trail making test is a test attention and executive function [24]. We used trail 5 of the Comprehensive Trail-Making Test (Pro-ed, Austin, Texas, USA), which is organized like the original trail making test, part B, developed in 1949, and added distracter circles [24]. In this test the subject must connect circled numbers and letters in alternating sequence (e.g. 1-A-2-B-3-C…). The time to finish the test was measured in seconds.

#### Stroop color and word test

This test measures selective attention and cognitive flexibility [25]. A Danish version of the standardized version by Golden [26] was used according to the test-manual. The test consists of three pages, each having 100 items presented in 5 columns. On the first page the words “red”, “green” and “blue” are printed in black and arranged randomly. On the second page “XXXX” is printed in red, green or blue and arranged randomly. On the last page words from the word page are printed in non-matching colors from the color page. On the first and the second page the subject is supposed to read loud the color of the items. On the word-color-page the subject must read the color of the ink for each item. Number of items during a 45 second period is recorded. Raw scores are presented.

### Neurophysiological Tests

#### Electroencephalography (EEG)

A neurophysiologist specialized in clinical EEG reading (TWK) analyzed the EEG data. Continuous 20-channel EEG was recorded. Data were filtered by a first order 0.53 Hz low cut filter and a first order 70 Hz high cut filter. All data were visually inspected in a linked earlobe reference montage. For each subject the electrode giving the best representation of the dominant activity was chosen for power spectral analysis, which was performed using fast fourier transformation (FFT) on 2.5 sec epochs (giving 0.4 Hz resolution in the FFT power spectrum). If a peak in the power spectrum could be identified in the range 4–13 Hz, it was extracted as representative for the dominant frequency.

### Biochemical Analyses

Plasma glucose concentrations were measured enzymatically at a central laboratory analyzer at Hillerød Hospital (COBAS INTEGRA, Roche, Basel, Switzerland). Bedside capillary blood glucose measurements used for monitoring during the experiments were done by a HemoCue Analyzer (HemoCue AB, Sweden). EPO concentrations were measured by the use of the Quantikine colorimetric sandwich IVD ELISA-technique (R&D Systems Europe, Abingdon, UK). Plasma glucagon and serum growth hormone concentrations were measured as previously described [27]. Blood for measurements of catecholamines was drawn in chilled tubes pre-added with a mixture of EGTA, glutathione and NaOH to prevent catecholamine degradation. Levels of catecholamines were determined with a commercial ELISA kit (BA E-5100, Labor Diagnostika Nord GmbH & Co, Nordhorn, Germany). All samples were centrifuged and stored at −80°C immediately after blood was drawn, except for blood for measurements of electrolytes, hematological parameters and cortisol. These samples were placed in the refrigerator (4°C) and measured with routine methods.

### Statistics

Statistical analyses were performed using statistical software (PASW Statistics, version 18.0, Windows, formerly SPSS). Entry into the database of all data regarding the primary endpoint and spot tests of entry of glucose values were verified by the GCP Unit at the University of Copenhagen.

Initially, a Wilcoxon signed ranks test was done to determine if the number of subjects performing better on the EPO day than on the placebo day differed from chance. Then paired t-tests were done to compare baseline values of the different endpoints with hypoglycemic values. Thereafter, placebo-corrected delta values were calculated ((X-hypo_PLACEBO_ – X-baseline_PLACEBO_) minus (X-hypo_EPO_ – X-baseline_EPO_)) in tests of cognitive function and compared to zero in a one sample t-test.

To further assess the impact of EPO treatment on cognitive function, hypoglycemic symptoms and cortical activity (measured with EEG) during hypoglycemia a mixed general linear model (analysis of covariance (ANCOVA)) was used (SPSS: Analyze>Mixed Models>Linear>Continue). Analysis for each cognitive endpoint was done separately. Because cognitive baseline measures could potentially be of importance for cognitive measures during hypoglycemia and during the recovery period, baseline values were pre-specified as a covariate and a fixed factor. This makes the analysis reliable and statistically more powerful [28]. Since small differences in plasma glucose between subjects and day of experimentation may potentially affect cognitive function, plasma glucose during the particular test was also pre-specified as a covariate and a fixed factor. Treatment (placebo vs. EPO) was considered a fixed factor and patient number was a random factor.

Analysis of adrenaline, glucagon, cortisol and growth hormone concentrations were based on peak values during hypoglycemia and mean values during euglycemia. The impact of EPO treatment on hormone levels during hypoglycemia and during the recovery period was assessed using the ANCOVA model as mentioned above, except that glucose concentrations were not included as a covariate.

Plasma glucose levels during the hypoglycemic phase on the two days of experiments were compared using a paired t-test. All calculations regarding the primary endpoint, cognitive function (reaction time tests, the trail making test and the Stroop test), were done blinded. The assumption about normally distributed residuals was checked via visual inspection of residual histograms. If necessary, data were log10 transformed before testing and in the ANCOVA results were back-transformed to percentage. Level of statistical significance was <5% (two-sided).

## Results

### Glucose Concentrations and Glucose Infusion Rate (for details see Table S1)

The mean capillary glucose concentrations (SD) measured bedside during hypoglycemia and used for adjustments of glucose infusion rate were 2.2 (0.1) mmol/l on the EPO day and 2.2 (0.2) mmol/l on the placebo day (p = 0.83, paired t-test). During hypoglycemia, mean plasma glucose concentrations were 2.2 (0.3) mmol/l on the EPO day and 2.0 (0.3) mmol/l on the placebo day (p = 0.043). The mean glucose infusion rate at euglycemia (baseline) and during hypoglycemia did not differ on the EPO and placebo days. There were no differences between plasma glucose values during the different cognitive tests and EEG recordings on the EPO day and the placebo days, except for during the reaction time tests. No differences in nadir plasma glucose concentrations were found between the EPO and the placebo day.

### Cognitive Function (Table 2)

In RT2, nine of 11 subjects had a lower reaction time prolongation during hypoglycemia (from baseline) on the EPO day than on the placebo day (p = 0.021, Wilcoxon signed ranks test). Placebo-corrected delta values (RT2hypo_PLACEBO_ – RT2baseline_PLACEBO_ minus RT2hypo_EPO_ – RT2baseline_EPO_) showed a 63 (SD 73) msec faster reaction time on the EPO day than on the placebo day (p = 0.016 (one sample t-test, comparison with 0)). In the adjusted analysis, EPO-treatment was associated with a 66 (−117–−16) msec reduction in reaction time compared to treatment with placebo (p = 0.02) (Table 2). Nine of 11 subjects produced fewer errors during hypoglycemia (compared to baseline) after EPO treatment than after placebo treatment (p = 0.018). Placebo-corrected delta values showed 4.7 (4.8) less errors after EPO treatment (p = 0.008). In the adjusted analysis treatment with EPO was associated with 4.7 (−8.1–−1.3) less errors compared to treatment with placebo (p = 0.01). In the simple reaction time test (RT1), the trail making test and the Stroop test there were consistently small but insignificant improvements after EPO treatment as compared to placebo treatment in the non-adjusted and adjusted analyses. There was no effect of EPO compared to placebo on cognitive performance during the recovery period.

**Table 2:** Cognitive function, EEG and symptoms of hypoglycemia during experiments.

	Placebo				EPO				Effect of EPO	
	Baseline	Hypo	Δ%	p	Baseline	Hypo	Δ%	p	mean (C.I.)	p
**Cognitive function (SD)**										
RT1, milliseconds	467 (56)	562 (106)	20	0.005	465 (63)	536 (137)	15	0.025	−21 (−102–61)	0.58
RT2, milliseconds	585 (125)	764 (124)	31	<0.0001	575 (135)	691 (150)	20	0.001	−66 (−117–−16)	0.02
Errors RT1, number	0.2 (0.4)	2.3 (2.7)	1050	0.017*	0.4 (0.7)	1.8 (3.1)	350	0.17*	−0.2 (−2.0–1.6)	0.80
Errors RT2, number	4.0 (2.6)	15.0 (6.0)	275	<0.0001	3.9 (3.8)	10.1 (4.8)	159	0.0002	−4.7 (−8.1–−1.3)	0.01
Trail making test B, seconds	56 (25)	127 (81)	127	0.0001**	59 (25)	123 (92)	108	0.01**	−14 (−44–30)%	0.42**
Stroop Word, items completed	94 (21)	63 (17)	−33	<0.0001	92 (20)	64 (24)	−30	<0.0001	3.5 (−5.8–12.9)	0.44
Stroop Colour, items completed	69 (17)	48 (14)	−30	<0.0001	71 (16)	50 (18)	−30	0.0001	2.4 (−6.0–10.8)	0.53
Stroop Word/Colour, items completed	46 (15)	28 (11)	−39	<0.0001	47 (15)	31 (13)	−34	0.0004	2.9 (−4.1–9.9)	0.39
**Neurophysiological test (SD)**										
EEG, Hz	9.5 (1.0)	7.6 (1.7)	−20	0.014	9.3 (0.9)	7.7 (1.9)	−17	0.01	0.14 (−1.6–1.9)	0.87
**Symptoms (SD)**										
Autonomic symptoms (3–21 points)	3.0 (0.0)	7.1 (4.3)	137	0.01	3.0 (0.0)	5.5 (2.4)	83	0.006	−1.6 (−4.0–0.9)	0.18
Symptoms of cognitive dysfunction (6–42 points)	6.6 (1.3)	11.4 (4.8)	73	0.003**	7.0 (1.5)	10.4 (4.1)	49	0.002**	−12 (−32–15)%	0.32**
Neuroglycopenic symptoms (4–28 points)	7.9 (3.4)	11.8 (4.8)	49	0.023	7.7 (3.7)	11.8 (5.0)	53	0.016	0.1 (−1.6–1.7)	0.94
Symptoms, total (13–91 points)	17.5 (3.9)	30.3 (12.8)	73	0.006	17.7 (4.7)	27.7 (9.6)	56	0.005	−2.8 (−8.6–3.0)	0.30

Measures of cognitive function, EEG and hypoglycemic symptoms at baseline and during hypoglycemia six days after an intravenous injection of 40,000 IU of EPO or placebo in 11 patients with type 1 diabetes. Autonomic and neuroglycopenic symptoms and symptoms of cognitive dysfunction are presented (in brackets the possible minimum and maximum points). P-values refer to comparisons between baseline and hypoglycemic values. The adjusted effect of EPO compared to placebo is presented in the two columns to the right (ANCOVA). Abbreviations: RT = reaction time. EEG = electroencephalography. EPO = erythropoietin. ANCOVA = analysis of co-variance.

* = Wilcoxon signed ranks test.

** = calculated on logarithmic values (log10) and in the ANCOVA back-transformed to per cent.

### EEG (Table 2)

EEG frequency decreased during hypoglycemia, but was not affected by EPO treatment. There was no effect of EPO compared to placebo on EEG frequency during the recovery period.

### Hematological, Hormonal Counter Regulatory and Cardiovascular Parameters and Adverse Events

Appear from Table 3 and from Table S2. There was no difference in the hormonal counter regulatory reponse during hypoglycaemia between EPO and placebo treatment. No EPO-related adverse events were recorded.

**Table 3:** Counter-regulatory hormones.

	Placebo			EPO			Effect of EPO	
Hematological and cardiovascular parameters (SD)	Base	Hypo	p	Base	Hypo	p	mean (C.I.)	p
EPO, mU/ml	11.2 (3.1)	13.2 (3.3)	0.026	12.3 (3.1)	13.7 (3.2)	0.022	−0.4 (−1.4–0.6)	0.4
Reticulocyte count, 10^9^/l	52.6 (13.0)	56 (14)	0.017	103 (30)	110 (35)	0.011	−2 (−10–5)	0.54
Hemoglobin, mmol/l	8.7 (0.8)	8.9 (0.9)	0.003	9.1 (0.9)	9.3 (1.0)	<0.001	0.04 (−0.09–0.16)	0.55
Hematocrit, fraction	0.41 (0.03)	0.42 (0.03)	0.009	0.42 (0.03)	0.44 (0.03)	<0.001	0.003 (−0.004–0.01)	0.42
Thrombocyte count, 10^9^/l	249 (39)	276 (47)	0.002	259 (54)	291 (61)	<0.001	5 (−4–13)	0.24
Sodium, mmol/l	138 (3.1)	140 (0.7)	0.014	138 (3.9)	140 (3.9)	<0.001	0.09 (−0.1–0.2)	0.86
Potassium, mmol/l	4.1 (0.5)	4.0 (0.7)	0.41	4.0 (0.2)	3.9 (0.2)	0.07	0.02 (−0.1–0.2)	0.70
Systolic blood pressure, mmHg	120 (15)	142 (19)	0.001	123 (19)	140 (25)	0.026	−5 (−14-5)	0.29
Diastolic blood pressure, mmHg	74 (7)	67 (7)	0.002	71 (12)	68 (9)	0.47	−2 (−3–7)	0.42
Heart rate, beats/min	69 (14)	76 (13)	0.14	69 (13)	75 (13)	0.001	−1.2 (−8.6–6.3)	0.71
**Hormones (SD)**								
Adrenaline, ng/ml	0.06 (0.03)	0.57 (0.42)	0.002	0.04 (0.03)	0.48 (0.29)	<0.001	0.07 (−0.07–0.21)	0.31
Glucagon, pmol/l	4.4 (0.8)	8.5 (2.9)	0.001	3.9 (1.6)	8.3 (3.6)	0.001	−0.8 (−2.5–0.9)	0.31
Cortisol, nmol/l	395 (126)	699 (180)	0.003	342 (84)	679 (222)	0.006	−26 (−90–37)	0.36
*Growth hormone, ng/ml	2.5 (4.3)	27.0 (8.8)	<0.001	2.4 (2.8)	22(11)	<0.001	−11 (−33–19) %	0.39*

Hematological and cardiovascular parameters and counter-regulatory hormones at baseline and during hypoglycemia six days after an intravenous injection of 40,000 IU of EPO or placebo in 11 patients with type 1 diabetes. P-values refer to comparisons between baseline and hypoglycemic values in a paired t-test. The adjusted effect of EPO compared to placebo is presented in the two columns at the right (ANCOVA). The hypoglycemic values are all peak-values, except for potassium and diastolic blood pressure, which are nadir values. Abbreviations: EPO = erythropoietin. ANCOVA = analysis of co-variance.

* = calculated on logarithmic values (log10) and in the ANCOVA back-transformed to percent.

### CGM during the Study

There was no difference in the number of hypoglycaemic episodes (episodes with CGM-glucose below 3.5 mmol/l) or recording time in the hypoglycaemic range between the EPO days and placebo days or between the first and the second experimental period.

### Adverse Effects

No adverse effects were recorded during and after experiments.

## Discussion

In this study we tested if intravenous treatment with EPO ameliorates cognitive dysfunction during hypoglycemia in patients with type 1 diabetes. EPO treatment was associated with a significantly better performance of a complex reaction time task in nine of eleven subjects with a lower error rate and a lower reaction time prolongation (44% reduction in increment of errors and 35% reduction of prolongation of reaction time during hypoglycemia when compared to placebo). In other tests of cognitive function there were no significant improvements after EPO treatment versus placebo treatment (p-values = 0.39–0.80). Hence, EPO treatment was not associated with an overall beneficial effect on cognitive function during hypoglycemia, but only in a complex reaction time task requiring the use of sustained attention/working memory.

The strength of the study is its randomized, double-blind, placebo-controlled, cross-over design, which - in theory - reveals only the effect of EPO on predefined endpoints. The use of cross-over studies in the field of diabetes has been criticized, since diabetes is a chronic progressive disease. However, in the present study, the time period between the two experimental days is relatively short, thus making this potential problem negligible and at the same time probably long enough (at least 6 weeks) to eliminate any carry-over effect of EPO. Although a carry-over effect cannot be totally excluded, the randomized design minimizes this possible effect of EPO and other factors, e.g. a learning effect, contributing to a period effect. Another strength of the study is the use of the glucose clamp technique to induce hypoglycemia. This technique makes it possible to attain precise glucose levels during the hypoglycemic phase, which can be reproduced in another experiment. However, the mean hypoglycemic plasma glucose level at the two days of experiments was slightly higher at the EPO day despite similar insulin and glucose infusion rates. The reason for this is unclear and may be due to several factors, including the effect of EPO on blood rheology and a possible effect of EPO on measurement accuracy, but may as well be a result of chance. However, it cannot be excluded that the capillary glucose measurements may have introduced an unwelcome inaccuracy in the plasma glucose measurements. A final strength of the study is the pre-experiment use of CGM with a hypo-alarm switched on, which made it possible for the subjects to counteract pending hypoglycemia. In that way antecedent hypoglycemia before the experiments is avoided. Antecedent hypoglycemia attenuates the counter-regulatory response and may lead to cerebral adaptation to hypoglycemia [29]. The present study has limitations, which may influence the results. Since it is the first of its kind, the determination of timing and dosage of EPO injection in relation to induction of hypoglycemia has been done on an empirical basis supported by only one previous study [17]. Also, the limited number of subjects participating makes it difficult to demonstrate small differences of the effect of EPO. However, such small differences may be of limited clinical significance.

Recently, two randomized, double-blind studies assessed the effect of EPO treatment on cognitive function. They showed that 40,000 IE of intravenously administered EPO lead to better performance in healthy subjects in a test of verbal fluency 7 days after injection of EPO [17], [30] and that weekly intravenous injections of 40,000 IE of EPO lead to improved performance in tests of cognitive function in chronic schizophrenic patients [19]. The populations in the two studies are very different from our population making comparisons difficult. Our findings, however, support the results of these studies [17], [19] and add to the results from cellular and animal studies showing that EPO protects and enhances the function of cerebral cells lacking substrate [13]–[15], [31]. Moreover, a recent study by Silverstein and coworkers has shown that erythropoietin ameliorates hypoglycemic brain damage in rats [16].

Our study indicates that EPO treatment may enhance some mental processes during hypoglycemia. Conservation of speed of reaction during hypoglycemia may be relevant in daily life e.g. during driving a vehicle. However, chronic EPO treatment to patients with diabetes is not acceptable since it is associated with thromboembolic episodes and increased blood pressure. Therefore, new non-hematopoietic EPO analogues may be of potential benefit, since they possess the pleiotropic effects of EPO without increasing the production of blood cells [32], [33] and therefore potentially may help patients with recurrent hypoglycemia to avoid these undesirable and potentially dangerous episodes.

## Supporting Information

Table S1 **Plasma glucose concentrations during the experiments.**
(DOCX)

Table S2 **Hematological and cardiovascular parameters.**
(DOCX)

Checklist S1 **CONSORT Checklist.**
(DOCX)

Protocol S1 **Trial Protocol.**
(DOCX)

Protocol Danish S1 **Trial Protocol in Danish.**
(DOCX)
