# Supplementary material for: Influence of Erythropoietin on Cognitive Performance during Experimental Hypoglycemia in Patients with Type 1 Diabetes Mellitus: A Randomized Cross-Over Trial
Source: PLoS One. 2013 Apr 5;8(4):e59672. doi: 10.1371/journal.pone.0059672 (PMC3618268; doi:10.1371/journal.pone.0059672)
Supplement: Table S1 — Plasma glucose concentrations during the experiments. (DOCX) [file pone.0059672.s001.docx]

**Table S1: Plasma glucose concentrations during the experiments**

|  | **Glucose concentration (SD)** | |  |
| --- | --- | --- | --- |
|  | Placebo day | EPO day | p |
| **Glycemic level at arrival to the laboratory** |  |  |  |
| Capillary glucose concentration, mmol/l | 10.9 (3.4) | 9.4 (3.4) | 0.37 |
| **Baseline (euglycemia)** |  |  |  |
| Mean plasma glucose, mmol/l | 4.5 (0.8) | 4.6 (0.8) | 0.67 |
| Glucose infusion rate, mmol x kg body weight^-1^ x min^-1^ | 0.015 (0.004) | 0.016 (0.005) | 0.64 |
| **Hypoglycemia** |  |  |  |
| Mean plasma glucose concentration, mmol/l | 2.0 (0.3) | 2.2 (0.3) | 0.04 |
| Nadir plasma glucose concentration, mmol/l | 1.8 (0.2) | 2.0 (0.3) | 0.23 |
| Glucose infusion rate, mmol x kg body weight^-1^ x min^-1^ | 0.0091 (0.004) | 0.0096 (0.004) | 0.57 |
| **Hypoglycemic plasma glucose during cognitive testing** |  |  |  |
| Reaction time test | 2.0 (0.3) | 2.2 (0.4) | 0.04 |
| Trail making test | 2.1 (0.3) | 2.2 (0.3) | 0.09 |
| Stroop test | 2.1 (0.3) | 2.1 (0.3) | 0.65 |
| EEG recording | 2.0 (0.3) | 2.1 (0.3) | 0.20 |

Plasma glucose concentrations and glucose infusion rates at baseline, during hypoglycemia and during cognitive testing (during hypoglycemia). Mean (SD) values are presented. P-values refer to paired t-tests between the placebo and the EPO day. EEG = electroencephalography.
