# Supplementary material for: Influence of Erythropoietin on Cognitive Performance during Experimental Hypoglycemia in Patients with Type 1 Diabetes Mellitus: A Randomized Cross-Over Trial
Source: PLoS One. 2013 Apr 5;8(4):e59672. doi: 10.1371/journal.pone.0059672 (PMC3618268; doi:10.1371/journal.pone.0059672)
Supplement: Table S2 — Hematological and cardiovascular parameters. (DOCX) [file pone.0059672.s002.docx]

**Table S2: Hematological and cardiovascular parameters**

|  | Placebo | | | EPO | | |
| --- | --- | --- | --- | --- | --- | --- |
| Entity, unit (normal range) | Day of injection | Day of experiment | p | Day of injection | Day of Experiment | p |
| Erythropoietin, U/l (3 - 17) | 11.3 (3.6) | 11.2 (3.1) | 0.95 | 13.1 (4.1) | 12.3 (3.1) | 0.43 |
| Reticulocyte count, 10^9^/l (25 - 99) | 49 (18) | 53 (13) | 0.25 | 46 (14) | 103 (30) | <0.0001 |
| Hemoglobin, mmol/l (8.1 - 10.3) | 8.7 (0.8) | 8.7 (0.8) | 0.89 | 8.6 (0.8) | 9.1 (0.9) | 0.002 |
| Hematocrit, fraction (0.40 - 0.50) | 0.41 (0.03) | 0.41 (0.03) | 0.76 | 0.40 (0.03) | 0.42 (0.03) | 0.001 |
| Thrombocyte count, 10^9^/l (145-390) | 241 (39) | 249 (39) | 0.35 | 243 (50) | 259 (54) | 0.054 |
| Leukocyte count, 10^9^/l (3.5 - 8.8) | 6.6 (2.5) | 6.5 (2.3) | 0.72 | 6.8 (3.1) | 6.7 (2.1) | 0.89 |
| Sodium, mmol/l (137 – 144) | 138 (3.1) | 136 (3.2) | 0.02 | 138 (4.2) | 138 (3.6) | 0.81 |
| Potassium, mmol/l (3.3 – 4.6) | 4.1 (0.5) | 4.3 (0.3) | 0.1 | 4.0 (0.2) | 4.2 (0.2) | 0.18 |
| Systolic blood pressure, mmHg | 136 (24) | 139 (25) | 0.44 | 142 (17) | 138 (26) | 0.49 |
| Diastolic blood pressure, mmHg | 78 (10) | 81 (10) | 0.10 | 82 (8) | 80 (9) | 0.58 |
| Heart rate, beats/min | 76 (12) | 71 (16) | 0.12 | 74 (13) | 71 (13) | 0.20 |

Hematological and cardiovascular parameters before and six days after an intravenous injection of 40,000 IU of erythropoietin or placebo in 11 patients with type 1 diabetes. P-values refer to paired t-tests. SD in brackets. Normal range is indicated in the left column.
