# Supplementary material for: Influence of Erythropoietin on Cognitive Performance during Experimental Hypoglycemia in Patients with Type 1 Diabetes Mellitus: A Randomized Cross-Over Trial
Source: PLoS One. 2013 Apr 5;8(4):e59672. doi: 10.1371/journal.pone.0059672 (PMC3618268; doi:10.1371/journal.pone.0059672)
Supplement: Protocol S1 — Trial Protocol. (DOCX) [file pone.0059672.s004.docx]

**Protocol**

Influence of erythropoietin on cognitive performance during experimental hypoglycemia in patients with type 1 diabetes mellitus

**Introduction**

*Objective*

To clarify if treatment with erythropoietin may enhance cognitive function during hypoglycaemia in patients with type 1 diabetes complicated by recurrent episodes of severe hypoglycaemia and impaired hypoglycaemia awareness.

*Background*

Preservation of cognitive function during hypoglycaemic episodes is crucial to patients with insulin-treated diabetes in order to avoid severe hypoglycaemic events, accidents and death. Amelioration of hypoglycaemia-induced cerebral dysfunction is therefore desirable and erythropoietin (EPO) may contribute to this, since a neuroprotective role has emerged for EPO in conditions with impaired substrate availability (1;2).

EPO is produced in the brain along with its own receptor, especially in response to intracerebral metabolic stress such as acute brain hypoxia (3). A number of studies suggest that EPO in a paracrine brain-specific system may preserve cognitive function during hypoglycaemia: EPO seems to improve synaptic transmission during and after combined lack of glucose and hypoxia (4), to increase GLUT 1 dependent glucose transport (GLUT 1 is the glucose transporter of the blood brain barrier) (5), and intravenous injection of high doses of EPO can activate the brain (measured by functional magnetic resonance imaging (fMRI)) and improve cognitive function of healthy subjects (6;7). Furthermore, in rats EPO stimulates the release of neurotransmitters (8). Recent published results from our research group show that patients with type 1 diabetes and low EPO concentrations have diminished cognitive function (that is longer reaction time) during hypoglycaemia than patients with higher level of EPO (9).

Whether intravenously administered EPO improves cognitive function during hypoglycaemia, is uncertain. It has been demonstrated that recombinant human EPO can penetrate the blood brain barrier, even in situations where the blood brain barrier is intact (10;11). The time from intravenously administration of EPO to induction of changes in cognition is only sparsely studied. A recent study suggests that the effect of EPO on cognitive function sets in between 3 and 7 days (6).

*Aim*

The aim is to test the hypothesis that treatment with erythropoietin enhances cognitive function during hypoglycaemia in patients with type 1 diabetes complicated by recurrent episodes of severe hypoglycaemia and impaired hypoglycaemia awareness. If the hypothesis is verified, the study may potentially lead to the development of a new supplementary treatment principle in insulin-treated diabetes.

**Methods**

*Design*

Double-blinded, randomised, placebo-controlled, cross-over trial. Blood glucose will be controlled by the hyperinsulinaemic glucose clamp technique. Primary endpoint is cognitive function during hypoglycaemia. Secondary endpoints are hypoglycaemic symptoms and hormonal counter-regulatory response.

*Material*

Patients from our outpatient clinic (Endocrinology Section, Hillerød Hospital) and from the outpatient clinic on Steno Diabetes Center who fulfil the criteria of inclusion will be invited to participate. Invitation will be send by letters to patients with 2 or more episodes of severe hypoglycaemia (assessed in an epidemiological study later published as: Kristensen PL, et al. Insulin analogues and severe hypoglycaemia in type 1 diabetes. Diabetes Res Clin Pract 2011, 96:17–23.). Twelve subjects will be included.

*Criteria of inclusion*

• Type 1 diabetes (WHO 1999).

• 2 or more hypoglycaemic episodes within the last year.

• Impaired hypoglycaemia unawareness (as defined in Pedersen-Bjergaard et al (11)).

• Age > 18 years.

• Duration of diabetes > 5 years.

• Weight > 50 kg.

• No pregnancy and safe contraception, according to The Medical Products Agency

guidelines.

• Signed informed consent

*Criteria of exclusion*

• Pregnancy or breast-feeding.

• Allergy to the study medicine.

• Heart-failure (NYHA 2-4).

• Known ischaemic heart disease

• Epilepsy.

• Earlier deep vein thrombosis / pulmonary embolism.

• History of thrombocytosis.

• Use of beta-receptor antagonists.

• Visual disturbance.

• Earlier stroke.

• Treatment or previous treatment with epoetin.

• Plasma creatinine above 100 umol/l for men and 88 umol/l for women at the last blood test.

• Surgery with suspected blood loss within the last 6 weeks.

• Solid or haematological cancer.

• Treatment with cyclosporine (interaction with epoetin).

• Suspected non-compliance with the study.

*Criteria for cessation*

• Withdrawal of informed consent.

• Pregnancy.

• Safety problems as assessed by the investigator.

• Non-compliance with the protocol as estimated by the investigator.

• Surgery with documented loss of blood before visit 3.

• Haemoglobin level below 7 or above 11 mmol/l in blood test taken at the Information visit.

• Thrombocytosis.

• Potassium > 5 mmol/l.

Patients who fail to finish the study will be replaced if possible.

**Description of the trial visits**

*Information visit*

The patient is informed about the trial. If the patient wants to participate, the informed consent form will be signed by the patient and the investigator. Criteria for inclusion and exclusion will be reviewed. Blood samples will be drawn. The patient is instructed to live as normally as possible and to avoid any vigorous exercise and the use of alcohol in the weeks before the experiments.

*Visit 1 (day 0)*

- Blood samples from the Information visit are checked.

- Following information is gathered: Demographic information: sex, age, ethnicity, handedness. Diabetes information: Year of diagnoses of diabetes, background or proliferative retinopathy, microalbuminuria, diabetic nephropathy (normo-, micro- or macroalbuminuria), amputation, hypoglycaemia awareness (a. m. Pedersen-Bjergaard (12) and Gold (13)), symptoms of neuropathy, insulin doses.

Other history: Smoking (+/-), exercise, alcohol consumption, other diseases, medicine, fertility.

Examination: Height and weight (without shoes), blood pressure (after 10 minutes in sitting position, measured twice), pulse. Clinical signs of deep vein thrombosis will be reviewed. Assessment of beat-to-beat variation and neuropathy. A pregnancy test will be carried out in all women with child-bearing potential.

- A continuous glucose monitoring system (CGMS) will be mounted to monitor the interstitial glucose concentration and the hypoglycaemic exposure from Visit 1 to Visit 2 and to ensure that the participant has not had hypoglycaemia in the night before the experiment.

- Randomization: 40.000 IU of Eprex® or placebo is injected intravenously over five minutes.

- Training in cognition tests (see below) will be done to prevent *learner’s effect* at the following visits.

*Visit 1½ (day 3)*

- Takes place 3 days after Visit 1. The CGMS is controlled and the sensor is replaced.

- The brain function of the subject is tested with Trail Making B Test, Stroop Test and

California Cognitive Assessment Package (CalCAP).

*Visit 2 (day 6)*

- Subjects meet in the laboratory in the morning at 08:00. It is ensured that no nocturnal

hypoglycaemia has taken place (one or more CGMS glucose value < 3.5 mmol/l or one or more blood glucose value < 3.5 mmol/l) - otherwise the experiment is postponed for 6 weeks. The visit is also postponed if plasma glucose > 20 mmol/l.

- Two intravenous cannulae are inserted (one retrograde and as close to the arterial-venous anastomoses as possible and one for administration of glucose and insulin).

- Equipment to monitor blood pressure and pulse are mounted.

- Equipment for electroencephalographic (EEG) recording (EEG cap and two precordial ECG leads connected to a digital EEG recorder) are mounted by two trained EEG-technicians.

- Using the hyperinsulinaemic glucose clamp technique a stable plasma glucose of 5-5.5 mmol/l is established. After 30 minutes of euglycaemia the plasma glucose is lowered to 2.0-2.2 mmol/l and kept stable at this level, while cognitive function tasks are conducted, EEG is recorded and blood samples are taken. The venous blood is arterialised by hot pads on the hand and forearm. The duration of hypoglycaemia is about 1-1 ½ hours. Then blood glucose is adjusted to 5-5.5 mmol/l, which is held for 30 minutes. The experiment is then stopped. The investigator will interrupt the experiment on subject’s request or if serious symptoms of severe hypoglycaemia occur. Glucose (10%) for intravenous use and juice will be ready during the trial.

Primary endpoints: Assessment of cognitive function by Trail Making B test, Stroop Test and CalCAP and the brain's spontaneous electrical activity measured by EEG. EEG will be analysed blinded after the trial by trained EEG analysts. Primary endpoints will be recorded before, during and after hypoglycaemia. The cognitive tests have been validated in a hypoglycaemic setting (14;15).

Secondary endpoints:

- Subjective hypoglycaemic symptoms evaluated using a validated questionnaire. The following symptoms will be assessed: Confusion, sweating, drowsiness, weakness, dizziness, sensation of warmth, palpitation, concentrating difficulties, double vision, blurred vision, hunger, nausea, anxiety, fatigue, tingling in the lips, trembling. The subject gives answer on a Likert scale of one to seven. For each symptom a score is achieved (16).

- Hormonal response and other substances (platelets, leukocytes, potassium, sodium, C-reactive protein (CRP), EPO, adrenaline, noradrenaline, glucagon, cortisol, growth hormone (GH), arterialised plasma glucose) will be measured in all the experimental cycles. Blood will be collected and stored at −80º C for later studies (proceeded by approval from the regional ethics committee).

*Visit 3 (6-10 weeks after the Visit 1)*

- A pregnancy test carried out on all women of child-bearing potential.

- Short physical examination (check for signs of deep venous thrombosis).

- The CGMS will be mounted

- An intravenous cannula is inserted for blood samples and injection of study medicine.

- Randomization.

- 40.000 IU of Eprex® or placebo is injected intravenously in over five minutes.

- Training in Trail Making B Test, Stroop Test and CalCAP.

*Visit the 3½ (3 days after Visit 3)*

- Takes place 3 days after Visit 3. The CGMS is controlled and the sensor is replaced.

- The brain function of the subject is tested with Trail Making B Test, Stroop Test and CalCAP.

*Visit 4 (6 days after Visit 3)*

Similar to Visit 2. Takes place at least 6 weeks after Visit 1. It is ensured that the patient since Visit 2 has not experienced loss of blood, for example due to surgery or trauma. At the end of the visit the subject will be asked to give a subjective assessment of when he received placebo or Eprex®. No further follow-up of the patient will take place.

*Randomization and blinding*

Subjects are assigned a sequential numbers starting with 1. Injection of placebo or study

medicine will be done by a doctor or nurse, which is employed in our research unit, but not involved in any phases of the study (third party). Injections will be done in a random, but balanced order and blinded to subject and investigator. Third party will record - secretly to the investigator - in what order the study medicine and placebo was given.

When all subjects have completed the study and all data are entered in the trial database, third party is asked to tell how the 24 injections were distributed into two groups, however, without providing the treatment attached to each injection. Subsequently, the data will be statistically processed. At the end of the analyses the code will be broken definitively.

Due to safety of subjects, the registration of what is injected can – if necessary – be seen by the investigator. Such a decision is taken by the primary investigator. Since Eprex® sometimes causes acute, short-term, flu-like symptoms, it is possible that the investigator may suspect what a given syringe contains.

*Study medicine*

Epoetin alfa

Eprex® (Janssen-Cilag A/S, Birkerød, Denmark), recombinant human erythropoietin, which is immunologically and biologically identical with the endogenous hormone, will be used. The dose is 40.000 IU. It will be administered intravenously approximately 6 days before induction of hypoglycaemia. Eprex® (and placebo) is stored in a refrigerator between 2 and 8° C. The temperature is monitored weekly.

*Insulin*

Human insulin (Actrapid, Novo Nordisk, Bagsværd, Denmark) is infused intravenously with a syringe pump. The dose is 1 IU/kg/min and the blood glucose concentration is adjusted with a variable infusion of glucose. The insulin is kept in a refrigerator between 2 and 8°C. The temperature is monitored weekly.

*Statistic Analysis*

Primary endpoints are cognitive function measured by validated cognitive function tests and the brain's spontaneous electrical activity measured by EEG. Secondary endpoints are symptoms of hypoglycaemia during hypoglycaemia (an arbitrary score) and hormonal counter-regulatory response. Data will be entered in a database and analysed using standard statistic software. The effect of intervention (Eprex® vs. placebo) on endpoints will be assessed by an analysis of co-variance (ANCOVA). Level of statistical significance will be 0.05 (two-sided). Power calculations show that a number of 10 subjects are needed (alfa = 0.05, beta = 0.20, minimal relevant difference in CalCAP test = 50 ms., SEM of reaction time during hypoglycaemia = 37.5 ms. Numbers are inserted in the formula shown below. = 7.85 according to a relevant table. Because of potential failure of participants to complete the study, 12 subjects will be included.

Formula used in cross-over design:

(reactiontime during hypoglycaemia)

2

*SEM*

⋅

⋅

×

=

*σ*

*relevant effect of treatment*

⋅

×

=

2

*δ*

 - in each ”group”.

*Approvals*

The study has been approved by the regional ethics committee (#H-A-2007-0116), the Danish Medicines Agency (#2007-00565141) and the Danish Data Protection Agency (#2007-41-1541). It is also registered at [www.clinicaltrials.com](http://www.clinicaltrials.com) (#NCT00615368). The study is conducted according to the ICH GCP guidelines.

*Ethical considerations*

Hypoglycaemia: During the experiments the subjects will be observed carefully by a doctor and a diabetes specialist nurse. The experiment will be stopped in case of severe cerebral dysfunction and on request from subject. One episode of hypoglycaemia at the intended glycaemic level will not lead to permanent injury. Other experiments using the same dose of same EPO as used in our study have shown that it is safe. If necessary the blinding of the study medication will be broken (17;18).

*Novelty and importance of this work*

Despite the development of new insulin analogues hypoglycaemia is still a significant problem in diabetes. In this research project we test a new potential treatment principle. If a beneficial effect of EPO on brain function during hypoglycaemia is demonstrated, the finding will open up the possibility of the development of a new supplementary treatment principle in insulin-treated diabetes. This will be of great value for patients and their relatives. A reduction in frequency of severe hypoglycaemia may lead to a better regulation of the glycaemic level, thereby reducing micro- and macrovascular complications.

**References**

1. Grasso G, Sfacteria A, Cerami A, Brines M (2004) Erythropoietin as a tissue-protective cytokine in brain injury: what do we know and where do we go? Neuroscientist. 10: 93-98

2. Brines M, Cerami A (2006) Discovering erythropoietin's extra-hematopoietic functions: biology and clinical promise. Kidney Int 70: 246-250

3. Hasselblatt M, Ehrenreich H, Siren AL (2006) The brain erythropoietin system and its potential for therapeutic exploitation in brain disease. J Neurosurg.Anesthesiol. 18: 132-138

4. Weber A, Maier RF, Hoffmann U, et al (2002) Erythropoietin improves synaptic transmission during and following ischemia in rat hippocampal slice cultures. Brain Res 958: 305-311

5. Ghosal J, Chakraborty M, Biswas T, Ganguly CK, Datta AG (1987) Effect of erythropoietin on the glucose transport of rat erythrocytes and bone marrow cells. Biochem.Med Metab Biol 38: 134-141

6. Miskowiak K, Inkster B, O'Sullivan U, Selvaraj S, Goodwin GM, Harmer CJ (2008) Differential effects of erythropoietin on neural and cognitive measures of executive function 3 and 7 days post-administration. Exp Brain Res 184: 313-321

7. Miskowiak K, Inkster B, Selvaraj S, Wise R, Goodwin GM, Harmer CJ (2008) Erythropoietin improves mood and modulates the cognitive and neural processing of emotion 3 days post administration. Neuropsychopharmacology 33: 611-618

8. Yamamoto M, Koshimura K, Kawaguchi M, Sohmiya M, Murakami Y, Kato Y (2000) Stimulating effect of erythropoietin on the release of dopamine and acetylcholine from the rat brain slice. Neurosci Lett 292: 131-133

9. Kristensen PL, Hoi-Hansen T, Olsen NV, Pedersen-Bjergaard U, Thorsteinsson B (2009) Erythropoietin during hypoglycaemia in type 1 diabetes: relation to basal renin-angiotensin system activity and cognitive function. Diabetes Res Clin Pract 85: 75-84

10. Juul SE, McPherson RJ, Farrell FX, Jolliffe L, Ness DJ, Gleason CA (2004) Erytropoietin concentrations in cerebrospinal fluid of nonhuman primates and fetal sheep following high-dose recombinant erythropoietin. Biol.Neonate 85: 138-144

11. Xenocostas A, Cheung WK, Farrell F, et al (2005) The pharmacokinetics of erythropoietin in the cerebrospinal fluid after intravenous administration of recombinant human erythropoietin. Eur.J.Clin.Pharmacol. 61: 189-195

12. Pedersen-Bjergaard U, Pramming S, Thorsteinsson B (2003) Recall of severe hypoglycaemia and self-estimated state of awareness in type 1 diabetes. Diabetes Metab Res Rev 19: 232-240

13. Gold AE, MacLeod KM, Frier BM (1994) Frequency of severe hypoglycemia in patients with type I diabetes with impaired awareness of hypoglycemia. Diabetes Care 17: 697-703

14. Pedersen-Bjergaard U, Thomsen CE, Hogenhaven H, et al (2008) Angiotensin-converting enzyme activity and cognitive impairment during hypoglycaemia in healthy humans. J Renin.Angiotensin.Aldosterone.Syst. 9: 37-48

15. Amiel SA (1998) Cognitive function testing in studies of acute hypoglycaemia: rights and wrongs? Diabetologia 41: 713-719

16. McCrimmon RJ, Deary IJ, Gold AE, et al (2003) Symptoms reported during experimental hypoglycaemia: effect of method of induction of hypoglycaemia and of diabetes per se. Diabet.Med. 20: 507-509

17. Kristensen J, Soegaard H, Maeng M, Rehling M, Nielsen TT (2006) Acute haemodynamic effects of erythropoietin alone and in combination with dopamine in a porcine model. Clin Physiol Funct.Imaging 26: 283-287

18. Ehrenreich H, Hasselblatt M, Dembowski C, et al (2002) Erythropoietin therapy for acute stroke is both safe and beneficial. Mol Med 8: 495-505
