# Supplementary material for: Influence of Erythropoietin on Cognitive Performance during Experimental Hypoglycemia in Patients with Type 1 Diabetes Mellitus: A Randomized Cross-Over Trial
Source: PLoS One. 2013 Apr 5;8(4):e59672. doi: 10.1371/journal.pone.0059672 (PMC3618268; doi:10.1371/journal.pone.0059672)
Supplement: Protocol Danish S1 — Trial Protocol in Danish. (DOCX) [file pone.0059672.s005.docx]

Titel: Erythropoietins betydning for den kognitive funktion hos diabetikere under eksperimentel hypoglykæmi.

**Kort titel:** ”HypoEpo Trial”.

**EudraCT nummer:** 2007-005651-41.

**Protokolkode:** 2007-185.

**Investigatorer:** Læge, klinisk assistent Peter Lommer Kristensen; Overlæge Ulrik Pedersen-Bjergaard; Overlæge, klinikchef, dr. med. Birger Thorsteinsson – alle fra Endokrinologisk Klinik, Hillerød Hospital og læge, dr. med. Lise Tarnow, Steno Diabetescenter.

**Sponsor:** Peter Lommer Kristensen.

**Kontakt adresse:** Læge, klinisk assistent Peter Lommer Kristensen, Hypoglykæmigruppen, Endokrinologisk Klinik, Hillerød Hospital, Helsevej 2, 3400 Hillerød. Telefon: 4829 7483. E-mail: pelk@noh.regionh.dk.

**Studiemedicin:** Epoetin alfa (Eprex, Janssen-Cilag A/S, Hammerbakken 19, 3460 Birkerød) og placebo.

**Laboratorier:**

- Klinisk Biokemisk Afdeling, Laboratoriediagnostisk Enhed, Hillerød Hospital, Helsevej 2, 3400 Hillerød.
- Department of Internal Medicine, University Hospital Maastricht, Debeyelaan 25, P.O. Box 5800, 6202 AZ Maastricht, The Netherlands v. Casper Schalkwijk.
- Neuroanæstesiologisk Klinik 2091, Neurocentret, Rigshospitalet v. Niels V. Olsen.
- Steno Diabetes Center, Niels Steensens Vej 2, 2820 Gentofte.
- Neurofysiologisk afdeling 3063, Rigshospitalet, Blegdamsvej 9, 2100 København v. Overlæge Troels Kjær (EEG tolkning).
- Medicinsk-Fysiologisk Institut, Panuminstituttet, København v. Jens Juul Holst.
- Center for Endokrinologi og Metabolisme (CEM), Herlev Hospital, Københavns Universitet v. Jens Faber.
- Medicinsk Endokrinologisk Afdeling M, Århus Sygehus v. Jan Frystyk.

**Monitor:** Københavns Universitetshospitals GCP-enhed, Gentofte Hospital, opg. 15, 2, Niels Andersens Vej 65, 2900 Hellerup.

**Etisk komité:** De Videnskabsetiske Komitéer for Region Hovedstaden, Regionsgården, Kongens Vænge 2, 3400 Hillerød.

**Apotek:** Hillerød Hospital, Hospitalsapoteket, Helsevej 2, DK-3400 Hillerød v. Sidse Dahlin.

**Signatur**

Peter Lommer Kristensen __________________________________ __________________

(protokolforfatter, sponsor, investigator) Underskrift Dato

**Bilag**

1: Plan over blodprøvetagning under eksperimentel hypoglykæmi.

2: Plan over blodprøvetagning under hele forsøget.

3: Retningslinier for mundtlig information.

4: Forsøgsrelaterede forkortelser.

5: Brev til potentielle forsøgspersoner – uden deltagerinformation (Hillerød version).

6: Brev til potentielle forsøgspersoner – uden deltagerinformation (Steno Diabetes Center version).

# Indholdsfortegnelse

[Indholdsfortegnelse 3](#_Toc195676119)

[Resumé 4](#_Toc195676120)

[Indledning 5](#_Toc195676121)

[Formål 6](#_Toc195676122)

[Materialer og metode 7](#_Toc195676123)

[Design 7](#_Toc195676124)

[Materiale/forsøgspersoner 7](#_Toc195676125)

[Inklusionskriterier 7](#_Toc195676126)

[Eksklusionskriterier 7](#_Toc195676127)

[Kriterier for studieophør 8](#_Toc195676128)

[Rekrutterings- og forsøgsplan 8](#_Toc195676129)

[Forsøgsbeskrivelse: 9](#_Toc195676130)

[Infomøde 9](#_Toc195676131)

[Besøg 1 (dag 0) 9](#_Toc195676132)

[Besøg 1½ (dag 3) 10](#_Toc195676133)

[Besøg 2 (dag 6) 10](#_Toc195676134)

[Besøg 3 (6-10 uger efter besøg 2 ) 11](#_Toc195676135)

[Besøg 3½ (dag 3 efter Besøg 3) 12](#_Toc195676136)

[Besøg 4 (dag 6 efter Besøg 3) 12](#_Toc195676137)

[Randomisering og blinding 13](#_Toc195676138)

[Studiemedicin 13](#_Toc195676139)

[Epoetin alfa 13](#_Toc195676140)

[Insulin 14](#_Toc195676141)

[Statistik 14](#_Toc195676142)

[Styrkeberegning 14](#_Toc195676143)

[Tidsplan 15](#_Toc195676144)

[Anmeldelser 15](#_Toc195676145)

[Kvalitetssikring 15](#_Toc195676146)

[Adverse events (AE) / serious adverse events (SAE) 16](#_Toc195676147)

[Dataindsamling og -behandling 17](#_Toc195676148)

[Projektets adresse 17](#_Toc195676149)

[Etiske overvejelser 17](#_Toc195676150)

[Bivirkninger til epoetin 18](#_Toc195676151)

[Øvrige ulemper for patienten 19](#_Toc195676152)

[Nytte ved forsøget 19](#_Toc195676153)

[Forsikring 19](#_Toc195676154)

[Økonomi 20](#_Toc195676155)

[Publikation 20](#_Toc195676156)

[Bilag 1 23](#_Toc195676157)

[Bilag 2 24](#_Toc195676158)

[Bilag 3 26](#_Toc195676159)

[Bilag 4 27](#_Toc195676160)

[Bilag 5 28](#_Toc195676161)

[Bilag 6 29](#_Toc195676162)

# Resumé

Introduktion:

Lavt blodsukker udløst af insulinbehandling (insulintilfælde) er et stort problem for mange diabetespatienter. Tilstanden er forbundet med nedsat livskvalitet og kan føre til hjerneskade og død. Under insulintilfælde reduceres hjernefunktionen så væsentligt, at patienten ikke kan genoprette blodsukkeret ved selv at indtage sukker, men har behov for hjælp fra andre. Erythropoietin (EPO) synes at beskytte hjernen ved hjerneskade, og der er teoretisk belæg for, at EPO kan bedre hjernefunktionen under insulintilfælde.

Formål:

Formålet med projektet er at undersøge, om EPO og placebo givet til diabetikere kan bedre hjernens funktion under kontrolleret lavt blodsukker. Primært endepunkt er kognitiv funktion. Sekundære endepunkter er graden af subjektive hypoglykæmiske symptomer og hormonalt respons.

Studie design:

Dobbeltblindet, randomiseret, placebokontrolleret overkrydsningsforsøg med kontrol af blodglukose ved hjælp af clamp-teknik. Kognitiv funktion måles ved hjælp af psykometriske tests.

Forsøgspersoner:

12 type 1 diabetes patienter med reduceret hypoglycaemia awareness og 2 eller flere insulintilfælde inden for det sidste år.

Interventioner:

EPO og placebo (isotonisk NaCl) givet intravenøst.

# Indledning

Normalt eller nærnormalt blodsukker er et af de vigtigste mål ved behandling af insulinkrævende sukkersyge, da et velkontrolleret blodsukker reducerer de alvorlige komplikationer til sukkersyge. Prisen for stram styring af blodsukkeret er desværre en øget risiko for lavt blodsukker, dvs. hypoglykæmi eller insulintilfælde (1). Hypoglykæmi er da også den hyppigste akutte bivirkning til insulinbehandling. Lette insulintilfælde opleves meget ofte i hverdagen og korrigeres nemt af diabetikeren, såfremt vedkommende har bevaret evnen til at mærke det lave blodsukkerniveau. Sværere insulintilfælde (= episoder med for lavt blodglukose, hvor diabetikeren er så omtåget, at assistance fra en anden person er påkrævet) skyldes ofte nedsat evne til at mærke det lave blodsukker (kaldes hypoglycaemia unawareness). Disse tilfælde, som er frygtet af patienterne og deres pårørende, er ledsaget af en ikke ubetydelig sygelighed og er i sjældne tilfælde dødeligt forløbende eller årsag til svær hjerneskade (2). De er endvidere en væsentlig barriere for god glykæmisk kontrol (3) og gentagne hypoglykæmiske episoder over tid medfører muligvis svækkelse af kognitiv formåen (4;5).

En mindre gruppe af diabetikere (ca. 20 %) rammes væsentligt oftere af insulintilfælde end gennemsnittet af diabetikere og årsagen hertil synes at være delvis genetisk bestemt (6). En egentlig forklaring på skævheden mangler. Denne undersøgelse skal kaste lys over om erythropoietin (EPO) som (forebyggende) tillægsbehandling til særligt udvalgte diabetespatienter kan vise sig at forhindre eller afsvække de skadelige effekter af hypoglykæmi.

*Erythropoietin*

Erythropoietin (EPO) er et glykoprotein, der hovedsageligt produceres i nyrerne. Traditionelt betragtes det som en hæmatopoietisk vækstfaktor, hvis produktion stimuleres af lav ilttension i nyrernes cortex (6). Det er en relativt ny opdagelse, at EPO også produceres i hjernen, hvor der også findes EPO receptorer (7). Der er tiltagende evidens for, at EPO virker cytoprotektivt ved en række patologiske tilstande. Bedst er det undersøgt ved traumatisk og anoxisk hjerneskade (apopleksi), hvor der synes at være en gevinst for de patienter eller dyr, der behandles med intravenøs EPO (8-10). EPO-behandling af anæmiske dialysepatienter er også vist at bedre den kognitive funktion signifikant (11), ligesom intravenøs indsprøjtning af høje doser EPO kan aktivere hjernen (målt ved hjælp af functional magnetic resonance imaging (fMRI)) og forbedre den kognitive funktion raske (12;13). I et randomiseret studie af effekten af EPO-behandling på intensivpatienter, hvor det primære endepunkt var antallet af blodtransfusioner, var der ingen forskel i EPO eller placebogruppen, men mortaliteten var mindre i patienter behandlet med EPO (14). EPO synes således at have også ikke hæmatopoietiske virkninger.

Den formodede cytoprotektive virkning af EPO synes at bero på EPOs antiapoptotiske egenskaber (15;16) og evnen til at facilitere tolerance overfor hypoxi (17) og desuden at mobilisere endotheliale progenitorceller og dermed fremme neovaskularisering (18). EPO er i en rottemodel vist at øge den synaptiske transmission i hjernen under og efter kombineret glukose- og oxygenmangel (19), og i en rottehjerne model stimulerer EPO frigørelse af dopamin og acetylcholin (20). Transport af glukose ind i ”fastende” erythrocytter stimuleres af EPO (21), hvilket i relation til neuroglykopeni er interessant, da erythrocytter og endothelceller i blod-hjerne-barrieren har samme transmembranøse og ikke-insulinafhængige glukosetransporter (GLUT1) (22). Til støtte for en sådan mekanisme, har endnu ikke publicerede resultater fra vores forskningsgruppe vist, at diabetespatienter med lave EPO koncentrationer har ringere kognitiv funktion (målt som længere reaktionstid) under hypoglykæmi end diabetespatienter med et højere EPO niveau.

Om endogen eller eksogen EPO virker neuroprotektivt og/eller forbedrer den kognitive funktion ved hypoglykæmi, er uvist, men ovenstående undersøgelser kunne tyde på det. Det er vist, at rekombinant human EPO kan penetrere blod-hjerne-barrieren, også i situationer, hvor blod-hjerne-barrieren er intakt (23;24). Tiden til den maksimale koncentration af EPO i cerebrospinalvæsken efter intravenøs indgift er varierende, men strækker sig i Xenocosta og medarbejderes arbejde fra 9-24 timer (23). Forsøg med rotter udsat for eksperimentel apopleksi viser, at EPO givet fra 24 timer før til 3 timer efter apopleksien nedsætter nekrosevolumen i hjernevævet (25). Der er således grund til at tro, at forbehandling med EPO 15-20 timer forud for eksperimentel hypoglykæmi burde give mulighed for at EPO kan transporteres ind i CNS. Tiden fra administrationen af EPO til en efterfølgende induktion af ændringer i kognition er kun sparsomt undersøgt. Nylige resultater fra Mikowiak et al. tyder på at effekten af EPO i kognitive funktionsundersøgelser først indsætter efter ca. 7 dage (12).

# Formål

At undersøge om eksogen tilførsel af exogen erythropoietin (epoetin) bedrer den kognitive funktion under eksperimentel hypoglykæmi hos diabetikere med tendens til svær hypoglykæmi og hypoglycaemia unawareness.

# Materialer og metode

Design

Dobbeltblindet, randomiseret, placebokontrolleret overkrydsningsforsøg med kontrol af blodglukose ved hjælp af clamp-teknik. Primært endepunkt er kognitiv funktion målt ved hjælp af validerede psykometriske tests.

Materiale/forsøgspersoner

Der søges rekrutteret 12 type 1 diabetes patienter via ambulatoriet i Endokrinologisk Klinik 0652, Medicinsk Enhed, Hillerød Sygehus og via ambulatoriet på Steno Diabetes Center. I foråret 2007 er samtlige patienter i ovennævnte ambulatorier kontaktet med henblik på afklaring af deres hypoglykæmitendens i forbindelse med en anden videnskabelig undersøgelse (HypoAna, EudraCT: 2006-003630-15). Ca. 80 patienter i Hillerød og 340 patienter på Steno Diabetes Center havde to eller flere insulintilfælde inden for det sidste år. De patienter, der ikke ønskede at deltage i undersøgelsen (HypoAna) skal danne grundlag for brevinvitation til nærværende undersøgelse (se Bilag 5). Patienter, der svarer positivt tilbage, vil blive inviteret til et indledende informationsmøde, hvor patienten vil blive mundtligt informeret forud for underskrivelse af det informerede samtykke.

### Inklusionskriterier

- Type 1 diabetes.
- 2 eller flere hypoglykæmiske episoder inden for det sidste år.
- Impaired hypoglycaemia unawareness (defineret som i Pedersen-Bjergaard et al (26).
- Alder >18 år.
- Diabetesvarighed > 5 år.
- Vægt > 50 kg.
- Negativ graviditetstest og sikker antikonception iht. Lægemiddelstyrelsens retningslinier.
- Underskrevet informeret samtykke.

### Eksklusionskriterier

- Graviditet eller amning.
- Allergi overfor indholdsstof.
- Hjerteinsufficiens (NYHA 2-4).
- Kendt iskæmisk hjertesygdom (defineret som antianginøs behandling eller/og tidl. bypass-operation eller PCI).
- Epilepsi.
- Tidligere dyb vene trombose/lungeemboli.
- Anamnese med thrombocytose.
- Brug af betablokkere (slører symptomer på hypoglykæmi).
- Synsnedsættelse, der besværliggør kognitiv funktionsmåling.
- Tidligere apopleksi.
- Behandling eller tidligere behandling med epoetin.
- Nedsat nyrefunktion (plasma-kreatinin over 100 umol/l for mænd og 88 umol/l for kvinder ved sidste blodprøve).
- Operation med formodet blodtab inden for de sidste 6 uger.
- Konstateret solid eller hæmatologisk cancer.
- Ciclosporinbehandling (interaktion med epoetin).
- Mistanke om non-kompliance med forsøgsprotokollen.
- Forhold, der efter investigators skøn er uforenelige med deltagelse, fx hensynet til patientens sikkerhed.

### Kriterier for studieophør

- Tilbagetrækning af informeret samtykke.
- Graviditet.
- Sikkerhedshensyn vurderet af investigator.
- Non-compliance med forsøgsprotokollen skønnet af investigator.
- Operation med dokumenteret blodtab før besøg 3.
- Hæmoglobinkoncentration < 7 eller > 11 mmol/l taget ved blodprøve ved Infomøde.
- Thrombocyttal > 400x10^9^ taget ved blodprøve ved Infomøde.
- Kalium > 5 mmol/l taget ved blodprøve ved Infomøde.

Udgåede patienter vil blive forsøgt erstattet. Data på patienter, der ikke gennemfører studiet, vil ikke blive inkluderet i dataanalyserne.

## Rekrutterings- og forsøgsplan

- Invitation til forsøget pr. brev (inkl. forsøgsinformation) (se [Bilag 5](#_Bilag_5) og [Bilag 6](#_Bilag_6)). Patienten opfordres til at ringe til os ved interesse. Alternativt kan e-mailes til os.
- Telefonisk samtale. Der aftales tid til informationsmøde. Inklusionskriterier checkes.
- Besøg 0: Informationsmøde (se også [Bilag 3](#_Bilag_3)). Munder ud i informeret samtykke. Der tages sikkerhedsblodprøver (Se [Bilag 2](#_Bilag_2)).
- Besøg 1: Anamnese. CGMS monteres, blodprøver, indgift af EPO eller placebo.
- Besøg 1½: CGMS checkes og udskiftes. Måling af enkelte endepunkter.
- Besøg 2: Hypoglykæmi, måling af endepunkter.
- Besøg 3: CGMS monteres, blodprøver, indgift af EPO eller placebo.
- Besøg 3½: CGMS checkes og udskiftes. Måling af enkelte endepunkter.
- Besøg 4: Hypoglykæmi, måling af endepunkter.

## Forsøgsbeskrivelse:

### Infomøde

Informationsmøde. Patienten orienteres mundtligt om forsøget i rolige omgivelser. Patienten kan stille spørgsmål. Hvis patienten ønsker at deltage, underskrives det informerede samtykke af læge og patient. Inklusionskriterier og eksklusionskriterier gennemgås. Herefter tages blodprøver (se [Bilag 2](#_Bilag_2)). Der aftales datoer for de følgende forsøgsdage. Patienten instrueres i at leve så normalt som muligt samt undgå stærk fysisk aktivitet og alkohol i ugen op til forsøget. Tiden fra Besøg 0 til Besøg 1 forsøges at være så kort som muligt, dog mere end en dag. Såfremt patienten ønsker mere betænkningstid inden underskrivning gives det – dog må betænkningstiden ikke overskride 2 døgn uden at patienten igen informeres mundtligt i detaljer – dvs. inviteres til et nyt informationsmøde. Blodprøver tages i så fald først ved dette andet informationsmøde og først når patienten har givet mundtligt og skriftligt samtykke til at deltage i undersøgelsen.

### Besøg 1 (dag 0)

- Blodprøver fra infomøde kontrolleres.
- Demografiske oplysninger: Køn, alder, etnicitet.
- Diabetes oplysninger: Debutår, simplex eller proliferativ retinopati, mikroalbuminuri, diabetisk nefropati (normo-, mikro- eller makroalbuminuri), amputation, hypoglykæmi awareness (ad modum Pedersen-Bjergaard (26) og Gold (27)), symptomer på neuropati, insulindosering.
- Øvrig anamnese: Rygning (+/-), motion, alkoholforbrug, andre sygdomme, samtidig medicin, fertilitet.
- Objektiv undersøgelse: Højde og vægt (uden sko), blodtryk (efter 10 minutter i siddende stilling målt to gange med et automatisk blodtryksmåleapparat), puls. Undersøgelse af underben for kliniske tegn på dyb vene trombose.
- En graviditetstest gennemføres på alle kvinder i den fertile alder.
- Patienten får herefter monteret kontinuerlig glukosemonitoreringsenhed (CGMS) for at kunne sikre at: 1) Deltagerens blodglukose i tiden fra Besøg 1 til Besøg 2 er så stabilt som muligt og 2) deltageren ikke har haft hypoglykæmi natten før forsøget^[[1]](#footnote-1)^.
- Der foretages vibrationssansmåling og måling af beat-to-beat variation.
- Der anlægges venflon og tages blodprøver (se Bilag 2).
- Patienten randomiseres.
- 40.000 IE epoetin alfa eller placebo injiceres intravenøst i en venflon over fem minutter.
- Forsøgspersonen gøres herefter fortrolig med detaljerne i forsøgsforløbet den næste uge og gennemgår de forskellige kognitionstests flere gange (for at hindre ”learners-effekt” ved Besøg 1½ og Besøg 2).
- Kan herefter tage hjem.

### Besøg 1½ (dag 3)

- Finder sted 3 dage efter Besøg 1. CGMS’en vil blive kontrolleret og skiftet eftersom batteriet ikke holder mere end 3 døgn.
- Forsøgspersonernes hjernefunktion vil blive testet: Der benyttes ”Trail Making B”, ”Stroop Test” og California Cognitive Assessment Package (CalCAP).

### Besøg 2 (dag 6)

- Forsøgspersonerne møder fastende om morgenen dagen efter besøg 1, og det sikres ved hjælp af glukosemonitoren, at der ikke har været natlig hypoglykæmi (blod-glukose < 3.5 mmol/l) – ellers udskydes forsøget 6 uger. Forsøget udskydes ligeledes hvis plasmaglukose > 20 mmol/l.
- Der anlægges 2 venflon én til blodprøvetagning (denne anlægges om muligt retrogradt så tæt på A-V anastomoser som muligt) og én til indgift af glukose/insulin, og der påsættes udstyr til monitorering af blodtryk og puls.
- Ved hjælp af clampteknik etableres et stabilt blodglukose på 5-5.5 mmol/l. Efter i alt ca. 30 minutters euglykæmi sænkes blodglukose langsomt til 2.0-2.2 mmol/l og holdes stabilt på dette niveau, mens der gennemføres neuropsykologiske undersøgelser og tages blodprøver fra venflon (se blodprøveplan i bilag 1). Det venøse blod arterialiseres ved at forsøgspersonens hånd placeres i en varmekasse. Varigheden af hypoglykæmien er ca. 1-1½ time. Herefter justeres blodglukose til udgangsværdien på 5-5.5 mmol/l, som holdes 30 minutter. Forsøget stoppes herefter. Undersøgelsen kan undervejs afbrydes på forsøgspersonens opfordring eller hvis der kommer alvorlige symptomer på svær hypoglykæmi eller ved blodglukose < 1.5 mmol/l. Glukose (10 %) til intravenøs brug og juice vil ligge klar under forsøget.
- Primært endepunkt

Vurdering af den kognitive funktion gennemføres ved at lade forsøgspersonerne gennemgå en række tests, der alle er meget benyttet til måling af kognitiv funktion under hypoglykæmi. Det drejer sig om ”Four Choice Reaction Test” (FCRT), ”Trail Making B” og ”Stroop Test”. I fald FCRT ikke kan gennemføres benyttes i stedet California Cognitive Assessment Package (CalCAP). Herudover måles hjernens corticale aktivitet ved hjælp af elektroencefalografi (EEG). EEG analyseres blindet efter forsøget af trænede EEG-analytikere. Der måles før, under og efter hypoglykæmi.

- Sekundære endepunkter

Forsøgspersonens subjektive hypoglykæmiske symptomer vurderes ved hjælp af et spørgeskema, som forsøgspersonen selv udfylder. Der spørges til følgende symptomer: Forvirring, sveden, døsighed, svaghed, svimmelhed, varmefornemmelse, talebesvær, hjertebanken, koncentrationsbesvær, kuldefornemmelse, dobbeltsyn, sløret syn, sult, kvalme, angst, træthed, prikken i læber, rysten/sitren. Forsøgspersonen svarer på en skala fra et til syv. For hvert symptom opnår forsøgspersonen en score (28).

Hormonelt respons vil blive målt i alle forsøgets cykler (se Bilag 1). Der udtages endvidere biologisk materiale i form af blod, som fryses ned (biobank) med henblik på evt. senere undersøgelser (se afsnittet Dataindsamling og -behandling).

### Besøg 3 (6-10 uger efter besøg 2 )

- En graviditetstest gennemføres på alle kvinder i den fertile alder.
- Kort objektiv undersøgelse (lunge- og hjertestetoskopi og undersøgelse for tegn på dyb venetrombose).
- Patienten får herefter monteret kontinuerlig glukosemonitoreringsenhed (CGMS) for at kunne sikre at: 1) Deltagerens blodglukose i tiden fra Besøg 1 til Besøg 2 er så stabilt som muligt og 2) deltageren ikke har haft hypoglykæmi natten før forsøget^[[2]](#footnote-2)^.
- Der foretages vibrationssansmåling og måling af beat-to-beat variation.
- Der anlægges venflon.
- 40.000 IE epoetin alfa eller placebo injiceres intravenøst i en venflon. Behandlingsrækkefølgen fremgår af forsøgsjournal (CRF) for besøg 2.
- Forsøgspersonen gøres herefter fortrolig med detaljerne i forsøgsforløbet til Besøg 4 og gennemgår de forskellige kognitionstests flere gange (for at hindre en ”learners-effekt” ved besøg 3½ og besøg 4).
- Kan herefter tage hjem.

### Besøg 3½ (dag 3 efter Besøg 3)

- Finder sted 3 dage efter Besøg 3. CGMS’en vil blive kontrolleret og skiftet eftersom batteriet ikke holder mere end 3 døgn.
- Forsøgspersonernes hjernefunktion vil blive testet: Der benyttes ”Trail Making B”, ”Stroop Test” og California Cognitive Assessment Package (CalCAP).

### Besøg 4 (dag 6 efter Besøg 3)

Som besøg 2. Finder sted mindst 6 uger efter besøg 2. Det sikres at patienten siden sidste besøg ikke har haft et blodtab, fx i forbindelse med operation eller traume. På det tidspunkt er en eventuel injektion af epoetin ved Besøg 2 metaboliseret. Om epoietin har en effekt, der rækker mere end 6 uger på studiets endepunkter er uvist, men lidet sandsynligt. Forsøgspersonen vil efter forsøget blive bedt om at give sin egen subjektive vurdering af hvornår vedkommende fik placebo eller epoetin. Efter besøg 4 vil der ikke være nogen yderligere opfølgning på patienten ud over den almindelige ambulante kontrol. Dog vil patienten få udleveret blodprøveseddel til måling af Hb og thrombocytter. Målingerne vil blive checket af investigator. Har der været hændelser i forbindelse med forsøget vil disse, hvis det er relevant, blive fulgt til dørs af investigator efter besøget.

Randomisering og blinding

Patienterne tildeles fortløbende numre startende med 1. Forsøgsmedicinen og placebo ankommer ikke i forfyldte sprøjter, men opbevares i ampuller i køleskab. Forsøgsmedicinen og placebo skal således trækkes op i sprøjte kort før injektion. Klargøring af og indsprøjtning af forsøgsmedicinen eller placebo foretages af en læge eller sygeplejerske, der er tilknyttet forskningsenheden, men som ikke er involveret i nogle faser af forsøget. I tilfældig rækkefølge - og uden investigators, hjælpepersonalets og forsøgspersonens vidende - indsprøjter denne ”tredje part” placebo eller forsøgsmedicin ved besøg 1 og 3. ”Tredje part” registrerer også - uden investigators, hjælpepersonalets og forsøgspersonens vidende - i hvilken rækkefølge forsøgsmedicinen og placebo er sprøjtet ind i ”Hemmelig Bog”. Denne registrering vil blive placeret på overlæge Birger Thorsteinssons kontor og findes dér i hele forsøgsperioden.

Ved hjælp af denne metode har hverken forsøgspersonalet eller forsøgspersonen nogen viden om hvilken behandling der gives ved Besøg 1 og 3. Metoden sikrer, at alle patienter behandles med både Eprex og placebo i tilfældig, balanceret rækkefølge.

Når alle forsøgspersoner har gennemført studiet, og alle data er indtastet i forsøgets database vil ”tredje part” blive anmodet om at oplyse hvordan de 24 indsprøjtninger fordeler sig i to grupper, dog uden at oplyse hvilken behandling der knytter sig til den enkelte indsprøjtning. Herefter vil data blive statistisk bearbejdet. Ved afslutning af analyserne vil koden blive brudt endeligt.

Registreringen af randomiseringen vil blive brudt hvis det af sikkerhedsmæssige grunde skønnes nødvendigt. En sådan beslutning tages af sponsor. Eftersom epoetin undertiden forårsager ”beskedne lokale gener samt akutte, kortvarige influenzalignende symptomer, der svinder i løbet af få timer”^^[[3]](#footnote-3)^^, eksisterer den mulighed, at forsøgsleder og forsøgspersonale kan få mistanke (men ikke vished) om hvad en enkelt sprøjte har indeholdt.

## Studiemedicin

### Epoetin alfa

Der anvendes epoetin alfa (Eprex, Janssen-Cilag A/S, Hammerbakken 19, 3460 Birkerød), som er rekombinant humant erythropoietin, der svarer immunologisk og biologisk til det endogene hormon. Dosis er på 40.000 IU og gives intravenøst én gang ca. 17-19 timer før der induceres hypoglykæmi. Denne dosis er en anelse højere end i et studie med apoplektiske patienter, der viste at 33.000 IU / patient gentaget over tre dage og administreret intravenøst (i alt 100.000 IU / patient IV), var en sikker behandling med god effekt på infarktstørrelse og post-infarkt deficit (dog blev der her anvendt epoetin beta) (10). Der vælges intravenøs administration for at opnå højest mulig koncentration i blodbanen og derved også i hjernen (23;24). Epoetinen (og placebo) opbevares i et medicinkøleskab mellem 2 og 8 ºC. Temperaturen monitoreres ugentligt. Ved køleskabet findes temperaturlog. Trial Master File vil indeholder et regnskab over forsøgsmedicinen.

### Insulin

Som ”redskab” til at inducere hypoglykæmi anvendes human insulin (Actrapid, Novo Nordisk, Bagsværd, Danmark). Dosis afpasses efter behov (målværdi blodsukker: 2.0-2.2 mmol/l). Insulinen opbevares i et medicinkøleskab mellem 2 og 8 ºC.

Statistik

Primært endepunkt er kognitiv funktion målt ved hjælp af validerede psykometriske tests og EEG. Sekundære endepunkter er subjektive hypoglykæmisymptomer under hypoglykæmi (en arbitrær score) og modregulationsrespons, herunder VEGF-respons. Data vil blive indtastet i en database og analyseret ved hjælp af statistiksoftware (SPSS). Interventionens (epoetin vs. placebo) betydning for endepunkterne vil blive vurderet ved hjælp af en analyse af co-varians (ANCOVA) med baselineværdier for de psykometriske tests som covariat. Der vil ikke blive foretaget interimanalyser. Data fra alle forsøgspersoner, der gennemfører hele forsøget, vil indgå i den statistiske analyse. Signifikansniveauet er 0.05.

## Styrkeberegning

En beregning af det nødvendige antal forsøgspersoner til undersøgelsen bygger på to oplysninger: For det første den mindste klinisk relevante forkortelse af reaktionstiden ved epoetin-behandling sammenlignet med placebo. For det andet ”standard error of the mean” (SEM) af reaktionstiden under hypoglykæmi. En fastlæggelse af første faktor beror på et skøn og sættes i denne undersøgelse til 50 ms. Fra vores tidligere forsøg ved vi, at den anden faktor kan sættes til ca. 37.5 ms. Tallene sættes ind i følgende formel, der benyttes ved et overkrydsningsdesign:

Med et tosidet signifikansniveau på 0.05og en ”styrke” på 80 % (, dvs. sandsynligheden for at forkaste en hypotese, som er falsk) bliver = 7.85 (ifølge tabel).

og

Beregnede nødvendige antal forsøgspersoner bliver derfor:

 - i hver “gruppe”.

Der er således behov for ca. 10 forsøgspersoner, men med et muligt frafald på op til 20 procent af forsøgsdeltagerne forsøges rekrutteret **12** forsøgspersoner i alt.

# Tidsplan

Protokol skrevet færdig November 2007

Godkendelse fra etisk komite og Lægemiddelstyrrelsen Feb. 2008

Protokolamendment 2 afsendes April 2008

Godkendelse fra etisk komite og Lægemiddelstyrrelsen af amendment 2 Maj 2008

Inklusion af patienter fra Maj 2008

Studiet afsluttes Ultimo 2008

Data analyse Primo 2009

Præsentation 2009

Publikation 2009

# Anmeldelser

Forsøget anmeldes til den Regionale Videnskabsetiske Komité og Datatilsynet. Da der er tale om et lægemiddelstudie, vil det endvidere også blive anmeldt til lægemiddelstyrelsen, CTG ([www.clinicaltrials.com](http://www.clinicaltrials.com)) og EUDRACT ([www.eudract.emea.eu.int](http://www.eudract.emea.eu.int)).

# Kvalitetssikring

Studiet gennemføres i overensstemmelse med Helsinki Deklarationen (1964), ICH GCP guidelines og forsøgsprotokollen. Københavns Universitetshospitals GCP-enhed monitorerer undersøgelsen og overensstemmelse med eksisterende regelsæt, samt at der er konsistens mellem CRF og kildedata. GCP-enheden og forsøget kan som led i kvalitetssikring af GCP-enhederne blive genstand for ekstern audit. Tilladelse til, at tredje person kan få adgang til patientdata, indhentes sammen med det informerede samtykke. Investigator giver adgang til kildedata og studierelevante dokumenter i forbindelse med monitorering, auditering eller inspektion fra en etisk komité, Lægemiddelstyrelsen eller andre landes sundhedsmyndigheder.

# Adverse events (AE) / serious adverse events (SAE)

Data om adverse events vil blive indsamlet løbende under forsøget i forsøgspersonens CRF og vil blive rapporteret til den etiske komite og Lægemiddelstyrelsen i henhold til gældende lovgivning og ICH-GCP guidelines. En SAE defineres som enhver utilsigtet medicinsk hændelse, der resulterer i død, er livstruende, medfører indlæggelse eller forlængelse af indlæggelse, resulterer i blivende eller betydende invaliditet eller medfødte abnormiteter. Hypoglykæmi (og heraf følgende ændringer i diverse biomarkører) er en forventet hændelse i denne undersøgelse vil derfor ikke bliver rapporteret til myndighederne som en AE/SAE. Undtagelsen til denne regel, er hvis svær hypoglykæmi medfører indlæggelse i mere end 24 timer, invaliditet eller død. En planlagt indlæggelse vil ikke blive betragtet som en SAE. AE defineres som en utilsigtet og uønsket medicinsk hændelse der opstår i tidslig forbindelse med forsøget, men som ikke kan defineres som en SAE og som kan eller ikke kan skyldes forsøgsinterventionen. Abnorme laboratorieværdier er regnes dog ikke som AE medmindre de er behandlingskrævende. Kun utilsigtede hændelser, der finder sted fra forsøgets start (injektion af placebo/EPO) til 7 dage efter forsøgsinterventionen, regnes som AE/SAE’er. I tiden mellem de to behandlinger (behandling Besøg 2 og 4) vil utilsigtede hændelser ikke blive registreret – undtaget herfor er dog de 7 dage efter Besøg 2. Standardiserede skemaer til indberetning af AE og SAE vil blive udformet som led i CRF’en. Følgende informationer vil blive indsamlet om en hændelse: Beskrivelse af hændelse, start- og slut-dato, intensitet af bivirkningen (mild/moderat/svær), formodet relation til studiemedicin, handling i forhold til hændelsen, handling i forhold til hændelsen. Ansvaret for alle indberetninger er Peter Lommer Kristensens. Skemaer til indberetning til myndigheder findes i forsøgets Trial Master File.

Forsøget vil blive ”sat på pause”, hvis sponsor finder det sandsynligt at en SAE kan relateres udelukkende til indgift af forsøgsmedicinen (epoetin alfa). Forsøget kan sættes i gang igen, hvis mistanken herfor bortfalder efter nærmere analyse. I tilfælde af en AE eller SAE vil patientens tilstand blive fulgt indtil patienten er kommet under relevant behandling/udredning hos praktiserende læge/sygehus/praktiserende speciallæge/anden relevant terapeut.

For bedre at kunne opdage en evt. bivirkning til injektion af epoetin, indskydes minimum en dag mellem de første fire forsøgsdeltagere. Såfremt disse forsøg ikke giver mistanke om svære bivirkninger, kan der gennemføres et forsøg pr. dag. Lægemiddelstyrelsen og Videnskabsetisk Komite vil efter forsøgets afslutning få en samlet rapport over hændelser og ikke en årlig rapport eftersom forsøget varer mindre end 1 år.

# Dataindsamling og -behandling

Kildedata vil være at finde i både forsøgspersonernes journaler og i de enkelte forsøgspersoners CRF. CRF’erne opbevares på et aflåst kontor. Disse data danner grundlag for forsøgets database, der etableres i computerprogrammet SPSS. Databasen vil befinde sig på Peter Lommer Kristensens personlige drev (H-drevet, hvortil afgang kun gives ved brug af et unikt password og brugernavn), som er en del af Hillerød Hospitals computernetværk. Data vil blive opbevaret efter Lov om behandling af personoplysninger og Sundhedsloven. Dataansvarlig er Peter Lommer Kristensen. Datavariable er nævnt i afsnittet om besøg 1. Data vedrørende forsøgets primære endepunkt indtastes dobbelt. En præcis liste over kildedatas placering findes i forsøgets Trial Master File. Der etableres en biobank bestående af blod, således at senere analyser af relevante nye markører kan udføres uden yderligere ulempe for de deltagende patienter efter godkendelse i den videnskabsetiske komité. En log over biobank-materiale vil blive oprettet. Biobankmateriale nedfrosset ved -80º C og data gemmes i 10 år og behandles fortroligt. I forbindelse med analyse af blodet kan det komme på tale at sende blodet til udenlandske laboratorier (inden for EU). Der er ikke planlagt udførsel af biologisk materiale til lande uden for EU.

# Projektets adresse

Projektet gennemføres i: Hypoglykæmigruppen, Endokrinologisk klinik 0652, Hillerød Hospital, Helsevej 2, 3400 Hillerød.

# Etiske overvejelser

Studiet vil blive anmeldt til det videnskabsetiske komitésystem og Lægemiddelstyrelsen. Rekruttering af deltagere vil afvente en tilladelse derfra. Deltagere vil modtage skriftlig information og mundtlig information (Se [Bilag 3](#_Bilag_3)). Det fremgår af den skriftlige information, at deltagelse er frivillig og ikke vil have indflydelse på fremtidige ambulant kontrol og behandling. Deltagelse er afhængig af afgivelse af skriftligt og mundtligt informeret samtykke, som kan trækkes tilbage på et hvilket som helst tidspunkt under studiet. Patienter, der ikke selvstændigt kan give informeret samtykke, vil ikke blive inviteret til at deltage. Såfremt forsøgspersonen ønsker yderligere oplysninger om projektet (fx mere detaljerede oplysninger om protokollen) gives disse umiddelbart af investigator/projektsygeplejerske. Ved yderligere behov henvises til klinikchef Birger Thorsteinsson. Under den hypoglykæmiske clamp vil patienten være under minutiøs observation af et team bestående af læge og en trænet diabetessygeplejerske, som løbende vurderer udvikling af kliniske tegn på svær hypoglykæmi. Glukose (10 %) til intravenøs brug vil ligge klar under forsøget. Undersøgelsen afbrydes med peroral glukoseindgift eller intravenøs glukoseinjektion ved begyndende kliniske tegn på svær cerebral dysfunktion, eller senest hvis blodglukose er < 1.5 mmol/l eller på patientens opfordring i øvrigt. En episode med hypoglykæmi på dette niveau er ikke forbundet med målbar risiko for pådragelse af varige mén hos i øvrigt raske diabetikere, men i vores tidligere forsøg har patienterne beskrevet træthed efter hypoglykæmi. I forbindelse med hypoglykæmi kan der opstå ubehag i form af svedeture, tremor, sult og almen følelse af ubehag. Intravenøs injektion med 33.000 IE epoetin beta gentaget tre dage i træk (kumuleret dosis ca. 100.000 IE) er i et tidligere forsøg med apoplektiske patienter beskrevet som sikkert (10). Forsøg med intravenøse doser af epoetin beta på op til 70.000 IE om måneden til raske voksne individer, har vist sig at være sikkert (29). I forsøg med primater er der tidligere givet væsentligt højere doser (5000 IU/kg.) uden at der er konstateret bivirkninger (24). Kristensen og medarbejdere (30) benyttede en epoetin beta dosis på 35.000 IE til grise med en kropsvægt som voksne mennesker (75 kg.) uden at kunne konstatere nogen hæmodynamiske ændringer.

Bivirkninger til epoetin

Uddrag fra Lægemiddelkatalogets ([www.medicin.dk](http://www.medicin.dk)) afsnit om erythropoietiske vækstfaktorer:

”I forbindelse med injektion af erythropoietin kan der optræde *beskedne lokale gener* samt akutte, kortvarige influenzalignende symptomer, der svinder i løbet af få timer. Arteriel hypertension ses. Generaliserede *epileptiske anfald* er i få tilfælde iagttaget hos patienter med hypertension, hos hvem hæmoglobinkoncentrationen var kommet op på det tilstræbte niveau eller derover, eller hvor anæmikorrektionen blev foretaget for hurtigt. *Arterielle og venøse tromboser* er beskrevet (AV-fistel, transplanterede nyrer, cerebrale tromboser). Den forbigående *stigning i trombocyttallet* ligger sædvanligvis inden for normalområdet, men trombocytose er set, og trombocyttallet skal kontrolleres de første uger under behandlingen. Stigende azotæmiparametre (kalium, phosphat, kreatinin, carbamid) kan ses under en hidtil sufficient dialysebehandling. *Pure Red Cell Aplasia* er en meget sjælden bivirkning forårsaget af anti-EPO antistof. Det er især set i relation til subkutan behandling med epoetin alfa. Undersøgelser tyder på, at de beskrevne tilfælde skal sættes i relation til udækkede gummistempler i opbevaringssprøjten.”

Det bemærkes, at teksten er præget af den kliniske situation, hvor epoetin administreres i månedsvis/årevis til patienter med kronisk nyresygdom, hæmatologisk eller onkologisk sygdom, hvilket også understreges i produktresuméet. Vi forventer ikke at se nogle af disse langtidseffekter efter en dosis erythropoietin. Der er en gang tidligere observeret anafylaktisk reaktion efter indgift af epoetin. Der vil derfor under indsprøjtning af forsøgsmedicinen være udstyr til at modgå anafylaksi til stede. Invasive procedurer omfatter anlæggelse af to venflon to gange og en CGMS-elektrode i underhuden to gange.

## Øvrige ulemper for patienten

Patienten skal deltage to hele dage (med ca. 6 ugers mellemrum), hvorfor forsøgspersonen evt. skal tage fri fra arbejde. Forsøgspersonen skal også møde op dagen før forsøget til montering af glukosesensor (CGMS) samt intravenøs injektion af EPO/placebo (2 x 1-2 timer), til udskiftning af CGMS tre dage efter (60 minutter) og til et kort informationsmøde (30 - 60 minutter). I forbindelse med blodprøvetagning tappes patienten for anslået 400 ml. blod over hele forsøget (minimum 6 uger). På forsøgsdagene skal forsøgspersonerne møde fastende. Hertil kommer ubehag/let smerte forbundet med blodprøver.

## Nytte ved forsøget

Den enkelte patient vil få et indtryk af deres hjernefunktion under hypoglykæmi - en viden, som muligvis kan hjælpe patienten til fremover bedre at opdage hypoglykæmi i tide. Undersøgelsens formål er at undersøge om behandling med industrielt fremstillet erythropoietin vil kunne bedre den kognitive funktion under hypoglykæmi. Dette er ikke undersøgt tidligere. Der er endvidere tale om forskning, der skal bibringe os viden om mekanismerne ved hypoglykæmi hos type 1 diabetikere. Hvis det viser sig at epoetin bedrer kognitionen under hypoglykæmi, kan det tænkes at epoetin behandling fremover hos en mindre gruppe af type 1 diabetikere, kan forebygge insulintilfælde. De medvirkende forsøgspersoner modtager 3000,- kr. for at deltage i forsøget. Ved aflyst besøg udbetales 500,- kr. yderligere. Dette beløb er en ulempegodtgørelse. Forsøgspersonernes eventuelle transportudgifter kan også blive kompenseret.

# Forsikring

Patienterne dækkes i henhold til gældende lovgivning af produkt-ansvars-forsikringen for studiemedicinen, samt lov om klage- og erstatningsadgang.

# Økonomi

Udgifter, budgetteret

| Anmeldelse til Lægemiddelstyrelsen | 7.010 |
| --- | --- |
| Løn PLK - 1 år | 484.000 |
| Løn diabetessygeplejerske - ½ år | 225.000 |
| Hormonanalyser (310 x 12 x 40) | 150.000 |
| Øvrige blodprøver | 25.000 |
| Epoetin alfa + placebo | 80.000 |
| Insulin | 1.000 |
| Infusionspumper og tilknyttet laboratorieudstyr | 50.000 |
| Kørselsgodtgørelse patienter. 300,- / patient | 7.200 |
| Ulempegodtgørelse (12x3000, -) | 36.000 |
| Administration, diverse | 75.000 |
| I alt | 1.140.210 |

Projektet er en del af Peter Lommer Kristensens Ph.d.-projekt, som finder sted i Forskningsgruppen Hypoglykæmigruppen på Hillerød Sygehus. Initiativet til projektet er taget af Peter Lommer Kristensen i samarbejde med tre af forskningsgruppens øvrige læger: klinikchef, overlæge, dr. med Birger Thorsteinsson, overlæge Ulrik Pedersen-Bjergaard og reservelæge, Ph.d. Thomas Høi-Hansen. Forskningsgruppen betaler lønudgifter i forbindelse med projektet. Øvrige udgifter søges dækket via private og offentlige fonde. Der er allerede fra denne side støttet med 483.000,- kr. En del af disse penge er øremærket til også andre aktiviteter i Ph.d.-projektet. Der forventes yderligere finansiering fra øvrige fonde. Ingen bevillinger er givet som personligt honorar til Peter Lommer Kristensen. Den forsøgsansvarlige er ikke knyttet til nogen private virksomheder eller fonde, som har en økonomisk interesse i forskningsprojektet.

# Publikation

Data ejes af investigatorerne. Data publiceres af studiets investigatorer i et internationalt tidsskrift med diabetes/fysiologi som interesseområde. Peter Lommer Kristensen er førsteforfatter. Undersøgelsens resultat vil blive offentliggjort uanset undersøgelsens udfald. En afsluttende rapport sendes til Lægemiddelstyrelsen inden 90 dage efter forsøgets afslutning og forsøgets resultater indsendes inden 1 år.

Reference List

1. The DCCT Research Group (1993) The effect of intensive treatment of diabetes on the development of long term complications in insulin-dependent diabetes mellitus. N.Engl.J.Med. 329: 977-986

2. Auer RN (2004) Hypoglycemic brain damage. Metab Brain Dis. 19: 169-175

3. Cryer PE (1994) Banting Lecture. Hypoglycemia: the limiting factor in the management of IDDM. Diabetes 43: 1378-1389

4. Gold AE, Deary IJ, Frier BM Recurrent severe hypoglycaemia and cognitive function in type 1 diabetes. Diabet.Med.%1993.Jul.;10(6.):503.-8.

5. Perros P, Deary IJ, Sellar RJ, Best JJ, Frier BM (1997) Brain abnormalities demonstrated by magnetic resonance imaging in adult IDDM patients with and without a history of recurrent severe hypoglycemia. Diabetes Care 20: 1013-1018

6. Ratcliffe PJ, Jones RW, Phillips RE, Nicholls LG, Bell JI (1990) Oxygen-dependent modulation of erythropoietin mRNA levels in isolated rat kidneys studied by RNase protection. J Exp Med 172: 657-660

7. Hasselblatt M, Ehrenreich H, Siren AL (2006) The brain erythropoietin system and its potential for therapeutic exploitation in brain disease. J Neurosurg.Anesthesiol. 18: 132-138

8. Brines M, Cerami A (6 A.D.) Discovering erythropoietin's extra-hematopoietic functions: biology and clinical promise. Kidney Int.%2006.Jul.;70.(2):246.-50.Epub.2006.May.31. Epub

9. Grasso G, Sfacteria A, Cerami A, Brines M Erythropoietin as a tissue-protective cytokine in brain injury: what do we know and where do we go? Neuroscientist.%2004.Apr;10(2):93.-8.

10. Ehrenreich H, Hasselblatt M, Dembowski C, et al (2002) Erythropoietin therapy for acute stroke is both safe and beneficial. Mol Med 8: 495-505

11. Marsh JT, Brown WS, Wolcott D, et al rHuEPO treatment improves brain and cognitive function of anemic dialysis patients. Kidney Int.%1991.Jan.;39.(1):155.-63.

12. Miskowiak K, Inkster B, O'Sullivan U, Selvaraj S, Goodwin GM, Harmer CJ (2008) Differential effects of erythropoietin on neural and cognitive measures of executive function 3 and 7 days post-administration. Exp Brain Res 184: 313-321

13. Miskowiak K, Inkster B, Selvaraj S, Wise R, Goodwin GM, Harmer CJ (2008) Erythropoietin improves mood and modulates the cognitive and neural processing of emotion 3 days post administration. Neuropsychopharmacology 33: 611-618

14. Corwin HL, Gettinger A, Fabian TC, et al (2007) Efficacy and safety of epoetin alfa in critically ill patients. N Engl J Med 357: 965-976

15. Ruscher K, Freyer D, Karsch M, et al Erythropoietin is a paracrine mediator of ischemic tolerance in the brain: evidence from an in vitro model. J.Neurosci.%2002.Dec.1;22.(23):10291.-301.

16. Calvillo L, Latini R, Kajstura J, et al Recombinant human erythropoietin protects the myocardium from ischemia-reperfusion injury and promotes beneficial remodeling. Proc.Natl.Acad.Sci.U.S.A.%2003.Apr 15.;100.(8.):4802.-6.Epub.2003.Mar.27. Epub

17. Prass K, Scharff A, Ruscher K, et al (2003) Hypoxia-induced stroke tolerance in the mouse is mediated by erythropoietin. Stroke 34: 1981-1986

18. Heeschen C, Aicher A, Lehmann R, et al Erythropoietin is a potent physiologic stimulus for endothelial progenitor cell mobilization. Blood.%2003.Aug.15.;102.(4):1340.-6.Epub.2003.Apr 17. Epub

19. Weber A, Maier RF, Hoffmann U, et al Erythropoietin improves synaptic transmission during and following ischemia in rat hippocampal slice cultures. Brain Res.%2002.Dec.27.;958.(2):305.-11.

20. Yamamoto M, Koshimura K, Kawaguchi M, Sohmiya M, Murakami Y, Kato Y (2006) Stimulating effect of erythropoietin on the release of dopamine and acetylcholine from the rat brain slice. Neurosci.Lett.2000 Oct 6;292.(2):131.-3.

21. Ghosal J, Chakraborty M, Biswas T, Ganguly CK, Datta AG (1987) Effect of erythropoietin on the glucose transport of rat erythrocytes and bone marrow cells. Biochem.Med.Metab Biol. 38: 134-141

22. Olson AL, Pessin JE (1996) Structure, function, and regulation of the mammalian facilitative glucose transporter gene family. Annu.Rev Nutr. 16: 235-256

23. Xenocostas A, Cheung WK, Farrell F, et al (2005) The pharmacokinetics of erythropoietin in the cerebrospinal fluid after intravenous administration of recombinant human erythropoietin. Eur.J.Clin.Pharmacol. 61: 189-195

24. Juul SE, McPherson RJ, Farrell FX, Jolliffe L, Ness DJ, Gleason CA (2004) Erytropoietin concentrations in cerebrospinal fluid of nonhuman primates and fetal sheep following high-dose recombinant erythropoietin. Biol.Neonate 85: 138-144

25. Brines ML, Ghezzi P, Keenan S, et al (6 A.D.) Erythropoietin crosses the blood-brain barrier to protect against experimental brain injury. Proc.Natl.Acad.Sci.U.S.A.2000.Sep.12.;97.(19.):10526.-31.

26. Pedersen-Bjergaard U, Pramming S, Thorsteinsson B (2003) Recall of severe hypoglycaemia and self-estimated state of awareness in type 1 diabetes. Diabetes Metab Res Rev 19: 232-240

27. Gold AE, MacLeod KM, Frier BM (1994) Frequency of severe hypoglycemia in patients with type I diabetes with impaired awareness of hypoglycemia. Diabetes Care 17: 697-703

28. McCrimmon RJ, Deary IJ, Gold AE, et al (2003) Symptoms reported during experimental hypoglycaemia: effect of method of induction of hypoglycaemia and of diabetes per se. Diabet.Med. 20: 507-509

29. Flaharty KK, Caro J, Erslev A, et al Pharmacokinetics and erythropoietic response to human recombinant erythropoietin in healthy men. Clin.Pharmacol.Ther.%1990.May.;47.(5):557.-64.

30. Kristensen J, Soegaard H, Maeng M, Rehling M, Nielsen TT Acute haemodynamic effects of erythropoietin alone and in combination with dopamine in a porcine model. Clin.Physiol Funct.Imaging.%2006.Sep.;26.(5):283.-7.

# Bilag 1

**Plan for måling af parameter under eksperimentel hypoglykæmi**^[[4]](#footnote-4)^

| CYKLUS | -2 | -1 | | | 1 | | | 2 | | | 3 | | | 4 | | | 5 | | |
| --- | --- | --- | --- | --- | --- | --- | --- | --- | --- | --- | --- | --- | --- | --- | --- | --- | --- | --- | --- |
| FASE | Klargøring | Justering  mod euglykæmi | | | Euglykæmi  *Baseline* | | | Justering  mod hypoglykæmi | | | Hypoglykæmi  *Stimulus* | | | Justering  mod euglykæmi | | | Recovery | | |
| BS  mmol/l | 5.0-20.0 | 5.0-20.0  Mål = 5.0-5.5 | | | 5.0-5.5 | | | Faldende  Mål = 2.0-2.2 | | | 2.0-2.2 | | | Stigende  Mål = 5.0-5.5 | | | 5.0-5.5 | | |
| TID  min | - | Variabelt | | | 30 (3x10) | | | Variabelt | | | 60 (3x20) | | | Variabelt | | | 30 (3x10) | | |
| Blodprøver^[[5]](#footnote-5)^ | - |  | | | x_1_ |  | x_1_ |  | - |  | x_1_ |  | x_1_ |  | - |  | x_1_ |  | x_1_ |
| BS | x | kontinuerligt | | | kontinuerligt | | | kontinuerligt | | | kontinuerligt | | | kontinuerligt | | | kontinuerligt | | |
| Kognitionstest | Repetition |  | - |  |  | x |  |  |  |  |  | x |  |  | - |  |  | x |  |
| Subj. sympt. | Præsentation |  | - |  | x_2_ |  |  |  |  |  | x_2_ |  |  |  | - |  | x_2_ |  |  |
| Puls + BT | x |  | - |  | x_3_ |  | x_2_ |  | x |  | x_3_ |  | x_2_ |  |  |  | x_3_ |  | x_2_ |

# Bilag 2

**Plan for blodprøvetagning under forsøget**

| **Blodprøver** | **Info-møde** | **Besøg 1** | **Besøg 2** | **Besøg 3** | **Besøg 4** | **Kontrol** | **Antal** |
| --- | --- | --- | --- | --- | --- | --- | --- |
|  |  | Intervention | HYPO | Intervention | HYPO |  |  |
| Hb | 1 | 1 | 6 | 1 | 6 | 1 | 16 |
| Reticulocytter |  | 1 | 6 | 1 | 6 |  | 14 |
| MCV |  | 1 |  | 1 |  |  | 2 |
| MCHC |  | 1 |  | 1 |  |  | 2 |
| Jern |  | 1 |  | 1 |  |  | 2 |
| Ferritin |  | 1 |  | 1 |  |  | 2 |
| Transferrin |  | 1 |  | 1 |  |  | 2 |
| Folat |  | 1 |  | 1 |  |  | 2 |
| Cobalamin |  | 1 |  | 1 |  |  | 2 |
| Trombocytter | 1 | 1 | 6 | 1 | 6 | 1 | 16 |
| Leukocytter |  | 1 | 6 | 1 | 6 |  | 14 |
| Kalium | 1 | 1 | 6 | 1 | 6 |  | 15 |
| Natrium |  | 1 | 6 | 1 | 6 |  | 14 |
| Creatinin | 1 | 1 |  | 1 |  |  | 3 |
| Carbamid |  | 1 |  | 1 |  |  | 2 |
| C-reaktivt protein (CRP) |  | 1 | 6 | 1 | 6 |  | 14 |
| HbA1c |  | 1 |  |  |  |  | 1 |
| C-peptid |  | 1 |  |  |  |  | 1 |
| EPO |  | 1 | 6 | 1 | 6 |  | 14 |
| VEGF |  | 1 | 6 | 1 | 6 |  | 14 |
| Endotheliale progenitor celler (EPC) |  |  | 2 |  | 2 |  | 4 |
| Plasma glucose |  | 1 |  | 1 |  |  | 2 |
| Arterialiseret glucose på Hemocue |  |  | 6 | 0 | 6 |  | 12 |
| Adrenalin |  |  | 6 | 0 | 6 |  | 12 |
| Noradrenalin |  |  | 6 | 0 | 6 |  | 12 |
| Glukagon |  |  | 6 | 0 | 6 |  | 12 |
| Kortisol |  |  | 6 | 0 | 6 |  | 12 |
| Væksthormon (GH) |  |  | 6 | 0 | 6 |  | 12 |
| Biobank |  | 1 | 6 | 1 | 6 |  | 14 |
| Graviditetstest |  | 1 | 0 | 1 |  |  | 2 |
| I alt pr. patient |  |  |  |  |  |  | 246 |

**Klinisk Biokemisk Afdeling, Hillerød Hospital:** Hb, reticulocytter, MCV, MCHC, jern, ferritin, transferrin, folat, cobalamin, thrombocytter, leukocytter, kalium, natrium, creatinin, carbamid, CRP, HbA1c, C-peptid, kortisol.

**Department of Internal Medicine, University Hospital Maastricht, Holland:** VEGF.

**Neuroanæstesiologisk Klinik 2091, Neurocentret, Rigshospitalet:** EPO.

**Steno Diabetes Center, Niels Steensens Vej 2, 2820 Gentofte:** EPC.

**Medicinsk-Fysiologisk Institut, Panuminstituttet, København (Jens Juul Holst):** Glucagon.

**Center for Endokrinologi og Metabolisme (CEM), Herlev Hospital, Københavns Universitet v. Jens Faber:** Adrenalin og noradrenalin.

**Medicinsk Endokrinologisk Afdeling M, Århus Sygehus v. Jan Frystyk:** Væksthormon.

# Bilag 3

**Retningslinier for mundtlig information.**

Den mundtlige information gives af investigator i samarbejde med en trænet forskningssygeplejerske og finder sted på et mindre kontor i Endokrinologisk Klinik, Nordsjællands Hospital – Hillerød. Kontoret er uforstyrret og der er plads til både forsøgsperson og en bisidder, som er velkommen. Tid og sted for den mundtlige informationssamtale er aftalt telefonisk eller pr. e-mail. Ved telefonsamtale eller i forbindelse med e-mail korrespondance vil retten til at medbringe en bisidder også blive nævnt.

Information vil blive givet i et letforståeligt sprog og der vil ved samtalen være god mulighed for at stille spørgsmål. Samtalen tager udgangspunkt i den skriftlige information og indeholder oplysninger om eventuelle forudsigelige risici, bivirkninger, komplikationer og ulemper, samt at der kan være uforudsigelige risici og belastninger knyttet til deltagelse i et biomedicinsk forskningsprojekt. Det vil også blive nævnt at vederlaget for deltagelse i forsøget er skattepligtigt. Investigator og/eller en trænet diabetes/forskningssygeplejerske er bemyndiget til at modtage samtykke.

Det er muligt, men ikke nødvendigt, at forsøgspersonen skriver under på det informerede samtykke i umiddelbar forlængelse af informationssamtalen, da der tages hensyn til at forsøgspersonen kan ønske betænkningstid. Såfremt patienten ønsker mere betænkningstid inden underskrivning gives det – dog må betænkningstiden ikke overskride 2 døgn uden at patienten igen informeres mundtligt i detaljer – dvs. inviteres til et nyt ”fuldt” informationsmøde.

Såfremt de første forsøg afslører bivirkninger, der kan relateres til epoetin alfa eller forskningsprojektets design ændres, vil kommende forsøgspersonerne blive informeret om dette.

Forsøgspersoner vil efter projektets afslutning blive informeret om forsøgsresultaterne.

Forsøgspersonernes ret til ikke at blive informeret om helbredstilstand og evt. disposition for sygdom vil blive respekteret.

# Bilag 4

**Forsøgsrelaterede forkortelser**

CGMS: Continuous Glucose Monitoring System.

EEG: Elektroencephalografi.

EPO: Erythropietin.

FCRT: Four Choice Reaction Test.

IE: Internationale Enheder.

IV: Intravenøs.

VEGF: Vascular Endothelial Growth Factor.

# Bilag 5

Til

***Kan EPO bedre hjernens funktion under et insulintilfælde?***

Vi skriver til dig for at spørge, om du vil deltage i et videnskabeligt forsøg. Med forsøget ønsker vi at undersøge, om EPO kan bedre type 1 diabetikeres hjernefunktion under insulintilfælde.

På de næste sider beskrives, hvad forsøget går ud på, og hvad deltagelse vil indebære for dig. Først når du er blevet informeret af en læge eller en sygeplejerske og har læst og forstået det følgende, kan du afgøre, om du vil deltage i forsøget. Tag dig god tid til at læse informationen og den vedlagte folder fra den Centrale Videnskabetiske Komité: ”Dine rettigheder som forsøgsperson i et biomedicinsk forskningsprojekt”.

Til den mundtlige informationssamtale er du velkommen til at medbringe en pårørende/ven, og der vil være tid til at stille spørgsmål. Du behøver ikke beslutte dig med det samme.

Det er frivilligt at være med i forsøget, og du kan når som helst trække dig ud af forsøget, uden at det vil påvirke din nuværende eller fremtidige kontrol og behandling.

Hvis du har spørgsmål til forsøget og/eller ønsker at deltage, er du velkommen til at ringe til læge Peter Lommer Kristensen på det direkte nummer **48 29 74 83.** Så kan vi aftale tid til et informationsmøde. Såfremt vi ikke hører fra dig, vil vi tillade os at kontakte dig efter nogle uger for at følge op på dette brev.

Med venlig hilsen

Peter Lommer Kristensen Birger Thorsteinsson

Læge, klinisk assistent Klinikchef, overlæge, dr. med

Endokrinologisk klinik

Hillerød Hospital

Helsevej 2

3400 Hillerød

Email: [pelk@hih.regionh.dk](mailto:pelk@hih.regionh.dk)

Telefon: 48 29 74 83

# Bilag 6

Til

***Kan EPO bedre hjernens funktion under et insulintilfælde?***

Vi skriver til dig for at spørge, om du vil deltage i et videnskabeligt forsøg. Med forsøget ønsker vi at undersøge, om EPO kan bedre type 1 diabetikeres hjernefunktion under insulintilfælde.

På de næste sider beskrives, hvad forsøget går ud på, og hvad deltagelse vil indebære for dig. Først når du er blevet informeret af en læge eller en sygeplejerske og har læst og forstået det følgende, kan du afgøre, om du vil deltage i forsøget. Tag dig god tid til at læse informationen og den vedlagte folder fra den Centrale Videnskabetiske Komité: ”Dine rettigheder som forsøgsperson i et biomedicinsk forskningsprojekt”.

Til den mundtlige informationssamtale er du velkommen til at medbringe en pårørende/ven, og der vil være tid til at stille spørgsmål. Du behøver ikke beslutte dig med det samme.

Det er frivilligt at være med i forsøget, og du kan når som helst trække dig ud af forsøget, uden at det vil påvirke din nuværende eller fremtidige kontrol og behandling.

Hvis du har spørgsmål til forsøget og/eller ønsker at deltage, er du velkommen til at ringe til læge Peter Lommer Kristensen på det direkte nummer **48 29 74 83.** Så kan vi aftale tid til et informationsmøde.

Såfremt vi ikke hører fra dig, vil vi tillade os at kontakte dig efter nogle uger for at følge op på dette brev.

Med venlig hilsen

Peter Lommer Kristensen Lise Tarnow

Læge, klinisk assistent Læge, dr. med, afdelingsleder

Endokrinologisk klinik

Hillerød Hospital

Helsevej 2

3400 Hillerød

Email: [pelk@hih.regionh.dk](mailto:pelk@hih.regionh.dk)

Telefon: 48 29 74 83

1. Der benyttes model med display, som alarmerer pt. ved blodglukose ≤ 4 mmol/l. I så fald kan pt. indtage mad og således undgå udvikling af hypoglykæmi. Hermed minimeres risikoen for at hypoglykæmi i dagene forud for Besøg 2 påvirker kognitionen og det hormonelle modregulationsrespons. I fald der har været hypoglykæmi i det sidste døgn forud for Besøg 2 udskydes dette besøg. Patienten skal fortsat være fastende ved Besøg 2. [↑](#footnote-ref-1)
2. Der benyttes model med display, som alarmerer pt. ved blodglukose ≤ 4 mmol/l. I så fald kan pt. indtage mad og således undgå udvikling af hypoglykæmi. Hermed minimeres risikoen for at hypoglykæmi i dagene forud for Besøg 2 påvirker kognitionen og det hormonelle modregulationsrespons. I fald der har været hypoglykæmi i det sidste døgn forud for Besøg 2 udskydes dette besøg. Patienten skal fortsat være fastende ved Besøg 2. [↑](#footnote-ref-2)
3. Fra [www.medicin.dk](http://www.medicin.dk). [↑](#footnote-ref-3)
4. x_1_, x_2_ og x_3_ benævner rækkefølgen af handlinger i samme delcycklus. SKAL LÆSES LODRET. [↑](#footnote-ref-4)
5. Trombocytter, Leukocytter, Kalium, Natrium, C-reaktivt protein (CRP), EPO, VEGF, Endotheliale progenitor celler (EPC), Adrenalin, Noradrenalin, Glukagon, Kortisol, Væksthormon (GH), Biobank. Arterialiseret glucose måles ca. hver 5. minut. [↑](#footnote-ref-5)
